# Computational analysis of expression of human embryonic stem cell-associated signatures in tumors

**DOI:** 10.1186/1756-0500-4-471

**Published:** 2011-10-31

**Authors:** Xiaosheng Wang

**Affiliations:** 1 Biometric Research Branch, National Cancer Institute, National Institutes of Health, Rockville, MD 20852, USA

## Abstract

**Background:**

The cancer stem cell model has been proposed based on the linkage between human embryonic stem cells and human cancer cells. However, the evidences supporting the cancer stem cell model remain to be collected. In this study, we extensively examined the expression of human embryonic stem cell-associated signatures including core genes, transcription factors, pathways and microRNAs in various cancers using the computational biology approach.

**Results:**

We used the class comparison analysis and survival analysis algorithms to identify differentially expressed genes and their associated transcription factors, pathways and microRNAs among normal vs. tumor or good prognosis vs. poor prognosis phenotypes classes based on numerous human cancer gene expression data. We found that most of the human embryonic stem cell- associated signatures were frequently identified in the analysis, suggesting a strong linkage between human embryonic stem cells and cancer cells.

**Conclusions:**

The present study revealed the close linkage between the human embryonic stem cell associated gene expression profiles and cancer-associated gene expression profiles, and therefore offered an indirect support for the cancer stem cell theory. However, many interest issues remain to be addressed further.

## Background

The development of human embryonic stem cell (hESC) is controlled by specific signatures, including specific transcription factors (TFs), pathways, microRNAs (miRNAs) and core genes. These signatures determine the self-renewal or differentiation fate of hESCs. Cancer is one of the developmental diseases. The initiation, proliferation and metastasis of cancer are often associated with the abnormalities of developmental signatures. Like hESCs, cancer cells are endowed with the ability to self-renew and proliferate indefinitely.

Based on accumulated evidence linking cancer cells to hESCs, some researchers proposed cancer stem cell (CSC) hypothesis [1]. A CSC is defined as "a cell within a tumor that possesses the capacity to self-renew and to cause the heterogeneous lineages of cancer cells that comprise the tumor [2] ". This hypothesis suggests that a small percentage of hESC-like CSCs are responsible for initiating and replenishing the tumor, and the dormant CSCs may account for cancer metastasis, chemoresistance and recurrence so that they become potential targets for improved cancer therapies. One type of evidence supporting the CSC model is the identification of surface markers of cancer-initiating cells (CICs; also known as cancer stem cells) in various human tumor types. Dick et al reported that only a subset of cells were able to transplant AML into recipient mice [3,4]. These tumorigenic cells were defined as CD34^+^CD38^-^, indicating a presence of CD34 proteins and a lack of CD38 proteins on their surface [5]. Dirks et al successfully isolated CSCs (CD133+ cells) from different phenotypes of brain tumors [6,7]. The CSCs were also identified in a list of the other tumor types including breast tumors [8], melanoma [9], ovarian cancer [10,11], prostate cancer [12], pancreatic cancer [13,14], sarcoma [15] and colon cancer [16,17]. Although the CSC theory is supported by some experimental evidences, much contention exists over whether these evidences are sufficiently valid or merely are some artifacts [18-21].

Some other types of evidence seems to lend support to the CSC theory, although they are not direct or absolutely convincing. For example, hESCs share cellular and molecular phenotypes with tumor cells and cancer cell lines [22]. Human induced pluripotent stem cells (HiPSCs) were first derived with four transcription factors: OCT4, SOX2, MYC and KLF4 [23] or OCT4, SOX2, NANOG, and LIN28 [24]. All these transcription factors have been reported to be highly expressed in various types of cancer [25-29]. Furthermore, silencing of tumor suppressor gene p53 significantly increased the reprogramming efficiency of human somatic cells [30]. Activation of telomerase is in part responsible for long lifespan of stem cells as well as anti-apoptosis of cancer cells [13,31-34]. Cell cycle regulation plays a critical role in both stem cells and cancer cells [35-39].

The linkage between hESC-specific gene expression profiles and cancer-specific gene expression profiles may provide evidence in support of the CSC model. To this end, many studies have identified hESC-associated gene expression signatures (hESCGESs) [40-44], and several studies have examined the expression of hESCGESs in human cancer [45-49]. In [45], the authors provided first clinical evidence for the implication of a "glioma stem cell" or "self-renewal" phenotype in treatment resistance of glioblastoma. In [46], the authors found the hESCGESs that distinguished primary from metastatic human germ cell tumors. In [47], the authors identified a subset of hESC-associated transcription regulators that were highly expressed in poorly differentiated tumors. In [48], the authors revealed that an increased expression of some hESCGESs identified poorly differentiated lung adenocarcinoma. In [49], the authors compared the expression of pluripotency factors OCT4, SOX2, KLF4 and MYC in 40 human tumor types to that of their normal tissue counterparts using publicly available gene expression data, and found significant overexpression of at least one out of them in 18 out of the 40 cancer types investigated. Furthermore, they found that these genes were associated with tumor progression or bad prognosis. All together, these studies revealed that "stemness" gene expression signatures were associated with tumor malignancies, and therefore might be informative molecular predictors of cancer therapy outcome [50].

In this study, we investigated the linkage between hESCGESs and tumor malignancies by an extensive examination of the expression of hESCGESs in various human tumor types. We used 51 publicly available gene expression datasets, which involve 23 human tumor types [51].

## Methods

### Identification of human stem cell-associated gene expression signatures

The self-renewal and differentiation of hESCs are controlled by hESC-specific signal molecules in a signaling-specific manner. Through a substantial survey of related literatures, we collected four types of hESCGESs: genes, pathways, TFs and miRNAs.

We collected 24 hESC-associated gene sets which were classified into five groups (Table 1 and Additional file 1, Table S1).

**Table 1:** hESC-associated gene sets

Group	Gene set	#Genes	Description
	hESC exp1	379	Overexpressed in hESCs according to 5 or more out of 20 profiling studies
	
	hESC exp2	40	Overexpressed in hESCs according to a meta-analysis of 8 profiling studies
	
	hESC exp3	48	Overexpressed in hESCs in at least 10 studies
	
	hESC exp4	30	Underexpressed in hESCs in at least 6 studies
	
	hESC exp5	189	TF genes in hESCs
	
hESC expressed	hESC exp6	44	Highly expressed in undifferentiated hESCs
	
	hESC exp7	994	High connectivity (≥ 500) in the global co-expression networks of hESCs
	
	hESC exp8	22	Candidate hESC markers
	
	hESC exp9	27	Differentially upregulated in hESCs
	
	hESC exp10	31	The most abundant transcripts expressed in hESCs
	
	hESC exp11	1, 000	Significantly identified in hESC lines by SAM analysis

	Nanog targets	988	Genes activated by Nanog in hESCs
	
	Oct4 targets	290	Genes activated by Oct4 in hESCs
	
NOS targets	Sox2 targets	734	Genes activated by Sox2 in hESCs
	
	NOS targets	179	Overlap of three above sets
	
	NOS TFs	37	Transcription regulators in NOS targets set
	
	NOS co-bound	353	Genes co-bound by Nanog, Oct4 and Sox2 in hESCs

	Suz12 targets	1, 040	Genes bound by Suz12
	
Polycomb targets	Eed targets	1, 066	Genes bound by Eed
	
	H3K27 bound	1, 121	Genes bound by H3K27
	
	PRC2 targets	654	Overlap of three above sets

Myc targets	Myc targets1	230	E-box-containing genes regulated by c-Myc in cultured cell lines
	
	Myc targets2	775	Genes commonly regulated by c-Myc and Max in a Burkitt's lymphoma cell line

P53 targets		35	Common gene lists regulated by p53 in mouse and human

A number of developmental signal pathways, such as Wnt, Notch, Hedgehog and Bmi-1, are necessary for regulation of stem cell self-renewal and differentiation. We identified 54 signal pathways as the hESC-associated pathway signatures (Table 2).

**Table 2:** hESC-associated signal pathways, TFs and miRNAs

Pathway	TF	miRNA
Activin	TP53	miR-143

AKT	MYC	miR-145

ALK	GATA4	miR-187

ATM	SMAD1	miR-296

BMI1	ESRRB	miR-301

BMP	SOX2	miR-21

Cell cycle	NANOG	let-7a

EGF/EGFR	KLF4	miR-371

ERBB2	MYB	miR-372

ERK	MYCN	miR-373

FGF	ZFX	miR-367

Glycolysis	STAT3	miR-302a

Hippo	ZIC3	miR-302a*

IGF	ZFP42	miR-302b

JAK/STAT	SALL4	miR-302b*

c-KIT	REST	miR-302c

Lefty	TCF3	miR-302c*

LIF	HOXB1	miR-302d

MAPK	HAND1	miR-200c

MEK/ERK	POU5F1	miR-222

NF-κB	SRY	

NHEJ/HR ^a^	TBX5	

Nodal	E2F4	

Notch	GATA6	

p53	PAX6	

PRC2	TCF4	

PDGF	FOXD3	

PI3K	CNOT3	

PTEN	ZEB2	

RAS	ESX1L	

RTK		

Hedgehog (SHH)		

Smad		

Stat3		

Telomerase		

TGFβ		

VEGF		

WNT		

^a ^NHEJ: non-homologous DNA end-joining; HR: homologous recombination

We identified 189 key TFs involved in regulation of hESC self-renewal and differentiation including three core TFs OCT4, SOX2 and NANOG with essential roles in the transcriptional control of the regulatory circuitry underlying pluripotency [43,52]. Table 2 lists 30 "critical" TFs. The complete TF list is presented in Additional file 2, Table S2.

Recent research indicates that miRNAs have an important role in regulating stem cell self-renewal and differentiation [53]. We identified 114 hESC-associated miRNAs. Table 2 lists one part of them. The complete miRNA list is presented in Additional file 3, Table S3.

### Identification of tumor-associated gene expression signatures

We identified differentially expressed genes among normal vs. tumor or good prognosis vs. poor prognosis phenotypes classes using univariate F-test for unpaired samples or t-test for paired samples at 0.05 significance level. This procedure was implemented with the class comparison between groups of arrays tool in BRB-ArrayTools, an integrated package developed by Simon et al for the visualization and statistical analysis of DNA microarray gene expression data [54]. The software can be freely downloaded from the website: http://linus.nci.nih.gov/BRB-ArrayTools.html.

We identified important pathways, TFs and miRNAs by analyzing gene sets for differential expression among pre-defined classes. The pre-defined phenotypes classes in the class comparison algorithm involved two types: normal vs. tumor and good prognosis vs. poor prognosis. The latter is concerned with tumor subtypes which exhibit different clinical outcome such as metastasis or not, relapse or disease free, drug or radio therapy sensitive or resistance etc., and different tumor progression grades. The LS or KS permutation test and Efron-Tibshirani's GSA maxmean test were used to determine the significant gene sets at 0.05 significance level. The pathways (BioCarta) related to the significant gene sets were identified. The TFs were identified by the gene sets, in each of which all genes were experimentally verified to be targets of the same transcription factor. Each miRNA potentially targeting all the genes in one of the gene sets was identified. The identification of important pathways, TFs and miRNAs was performed with the gene set expression class comparison tool in BRB-ArrayTools.

In addition, we used the survival analysis tool in BRB-ArrayTools to find genes, pathways, TFs and miRNAs related to survival for the partial datasets which provided related data. All the executive parameters were identical to those used in the class comparison.

We compared the identified gene sets, pathways, TFs and miRNAs to those in hESCGESs, and found their overlaps, respectively.

### Materials

We analyzed 51 human gene expression datasets involving 23 tumor types (Table 3). For each dataset, we carried out class comparison and/or survival analysis algorithm to identify informative genes, pathways, TFs and miRNAs. A total of 75 class comparison and survival analysis were carried out (Table 4). All the references relevant to Table 1, Table 2, Table 3 and Table 4 are presented in Additional file 4.

**Table 3:** Fifty-one human tumor gene expression datasets

Tumor Type	# Datasets
Bladder Cancer	1

Brain Cancer	6

Breast Cancer	5

Colon Cancer	1

Cervical Cancer	1

Embryonal Cancer	1

Esophageal Cancer	1

Gastric Cancer	2

Head and Neck Cancer	3

Leukemia	3

Liver Cancer	1

Lung Cancer	2

Lymphoma	4

Medulloblastoma	1

Melanoma	2

Mesothelioma	1

Ovarian Cancer	1

Pancreatic Cancer	1

Prostate Cancer	5

Renal Cancer	4

Soft Tissue Sarcoma	2

Thyroid Cancer	1

Uterine Leiomyoma	2

**Table 4:** Summary of the algorithms performed for all datasets

Algorithms		#Algorithms performed
Class comparison	normal vs. tumor	31
	
	good prognosis vs. poor prognosis	38

Survival analysis		6

## Results

### Overlaps between hESCGESs genes and tumor-associated genes

In the total of 75 class comparisons and survival analyses, we identified 72 sets of differentially expressed genes significant at 0.05 threshold level (Additional file 5, Table S4). We analyzed the overlap between each of the 72 gene sets and each of the 24 hESC-associated gene sets. We found that they have considerable overlaps. For example, all the 379 genes in the hESC exp1 gene sets of Table 1 appeared in at least one of the 72 differentially expressed gene sets (DEGSs). Among them, 308 genes appeared in 10 or more DEGSs, and 120 genes appeared in 20 or more DEGSs. The most frequently overlapping gene was MTHFD2 (methylenetetrahydrofolate dehydrogenase (NADP+ dependent) 2, methenyltetrahydrofolate cyclohydrolase), which occurred in one half the 72 DEGSs. The second most overlapping genes are MCM4 and MCM6 (34 overlaps), two members of the gene family encoding the mini chromosome maintenance complex. All the 40 genes in the hESC exp2 gene sets of Table 1 also occurred in at least one DEGS, and 26 genes occurred in no less than 10 DEGSs. Among them, MYBL2, a member of the MYB family of transcription factor genes involved in cell cycle progression, most frequently occurred in the DEGSs (31 times).

Table 5 gives the number of the genes which have 10 or more overlaps and the top 10 overlapping genes in each of the 24 hESC-associated gene sets, suggesting that a large proportion of the hESC-associated genes are also related to cancer. Gene function enrichment analysis suggests that a substantial portion of the genes listed in Table 5 are involved in cell cycle regulation, DNA damage repair and replication, apoptosis, development and differentiation, cell adhesion and TF activity (Table 6).

**Table 5:** Overlaps between the 24 hESC-associated gene sets and the 72 differentially expressed gene sets

Gene sets	**#Genes with 10 or more overlaps **^b^	Top 10 overlapping genes
hESC exp1	308 (81%)	MTHFD2, MCM4, MCM6, LGALS8, PPP2R1B, RFC4, GART, BUB1, LCK, PTPN2

hESC exp2	26 (65%)	MYBL2, EPHA1, ORC2, DTYMK, PRKD3, NCAPH, ETV4, DSCC1, CDC25A, PWP2

hESC exp3	38 (79%)	BUB1, DLGAP5, SLC16A1, USP9X, HSPA4, TERF1, PSIP1, PLA2G16, UGP2, BMPR1A

hESC exp4	29 (97%)	SPARC, COL1A2, COL3A1, COL1A1, CD47, COL5A2, KRT18, KRT8, LUM, COL6A3

hESC exp5	135 (71%)	TCF4, STAT1, GATA3, MAF, MYC, MYBL2, ILF3, SMAD4, FUBP1, GATA2

hESC exp6	28 (64%)	CD9, IL6ST, PTEN, EDNRB, KIT, NR5A2, IFITM2, CRABP2, NFYC, PODXL

hESC exp7	863 (87%)	GNAS, FN1, SPARC, MCM6, MCM4, TOP2A, COL1A2, COL3A1, RFC4, RAB31

hESC exp8	11 (50%)	FAS, CKS1B, GJA1, NPM1, TGIF1, HMGA1, DNMT3A, ERH, SOX2, DNMT3B

hESC exp9	21 (78%)	PCNA, CKS1B, FAS, TERF1, GJA1, CCNB1, NPM1, FZD7, SFRP1, HMGA1

hESC exp10	30 (97%)	PGK1, BAK1, HNRNPA1, EEF1A1, PPIA, GAPDH, GJA1, TMED2, EEF1B2, NPM1

hESC exp11	749 (75%)	PDE4DIP, FGFR2, CKS2, MTHFD2, SOX4, SLC2A3, STAT1, MAPK1, PSMB2, MCM4

Nanog targets	711 (72%)	CALD1, FGFR2, CKS2, TNPO1, KIAA0101, SPARC, TOP2A, ARHGAP1, B2M, VCAN

Oct4 targets	211 (73%)	FGFR2, TCF4, KIAA0101, TOP2A, PPP2R1B, TCF12, UBE2D3, RAB5A, HMGB2, PTPN2

Sox2 targets	542 (74%)	FGFR2, TOP2A, ARHGAP1, VCAN, PPP2R1B, UBE2D3, CBX3, RAB5A, PIK3R3, H2AFX

NOS targets	132 (74%)	FGFR2, TOP2A, PPP2R1B, UBE2D3, RAB5A, PTPN2, SET, FGFR1, BUB3, ADD3

NOS TFs	28 (76%)	STAT3, MYST3, IFI16, MLLT10, FOXO1, PHF17, ZFP36L1, TAF12, HHEX, ZEB2

NOS co-bound	199 (56%)	FGFR2, TOP2A, PPP2R1B, UBE2D3, RAB5A, PTPN2, SET, FGFR1, BUB3, BMI1

Suz12 targets	512 (49%)	PDE4DIP, BCL2, GNAS, PTGER3, CD44, RAB31, CYP1B1, EPHB1, GATA3, HLF

Eed targets	510 (48%)	PDE4DIP, BCL2, PTGER3, PRKCB, CD44, INPP4A, CYP1B1, EPHB1, GATA3, HLF

H3K27 bound	557 (50%)	PDE4DIP, BCL2, PTGER3, PRKCB, EPHB1, GATA3, NTRK2, CD47, GPD1L, NCAM1

PRC2 targets	397 (61%)	PDE4DIP, BCL2, PTGER3, EPHB1, GATA3, NTRK2, NCAM1, CACNA1D, GATA2, GPM6B

Myc targets1	204 (89%)	BCL2, MCM4, TGFB3, CCND2, APC, MUC1, TCF12, ENO1, APP, CSTB

Myc targets2	645 (83%)	CKS2, PPP2R1B, TCF12, UBE2D3, CDC25B, H2AFX, HLA-A, PDK3, PRKDC, TMF1

P53 targets	27 (77%)	GATM, MYH11, TP53, BTG2, INPP5D, MDM2, MYB, SPARCL1, PLK4, FMO5

^b ^The percentage of the overlapping gene number relative to the total gene number for each of the 24 hESC-associated gene sets is given in parenthesis.

**Table 6:** Functional categories of the genes listed in Table 5

Functional categories	Representative genes
Cell cycle regulation	ORC2, NCAPH, DSCC1, CDC25A, CDC25B, CKS2, CKS1B, BUB3, CCND2, CCNB1, DLGAP5

DNA repair/replication	TOP2A, MCM4, MCM6, RFC4, PCNA, PPIA, HMGB2, H2AFX, PRKDC

Apoptosis	FAS, BAK1, IFI16, PHF17, BTG2, PSMB2, BCL2, APC, TP53

Development/differentiation	ETV4, FGFR1, FGFR2, VCAN, STAT3, HHEX, EPHB1, NTRK2, GPM6B, TGFB3, BMI1, SOX2, DNMT3B, SOX4

Cell adhesion	VCAN, CD9, CD44, CD47, NCAM1, COL6A3, PODXL, FN1

TF activity	GATA2, TMF1, STAT1, STAT3, MAF, FUBP1, MYB, NFYC, TGIF1, SOX2, ZEB2, TAF12, ZFP36L1, MLLT10, MYST3, TCF4, TCF12, ETV4, MYBL2

**Figure 1:**
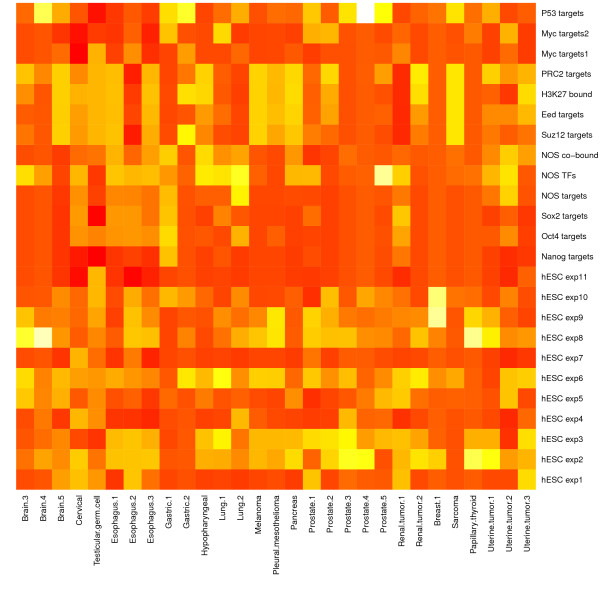
**Significance of overlap between hESC and tumor gene sets by normal vs. tumor class comparison**. The detailed description of all the datasets is provided in Additional file 6.

**Figure 2:**
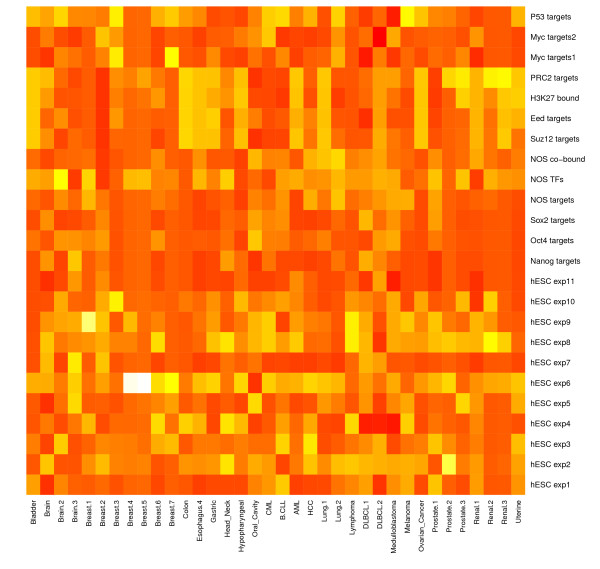
**Significance of overlap between hESC and tumor gene sets by good vs. poor prognosis class comparison**. The detailed description of all the datasets is provided in Additional file 6.

**Figure 3:**
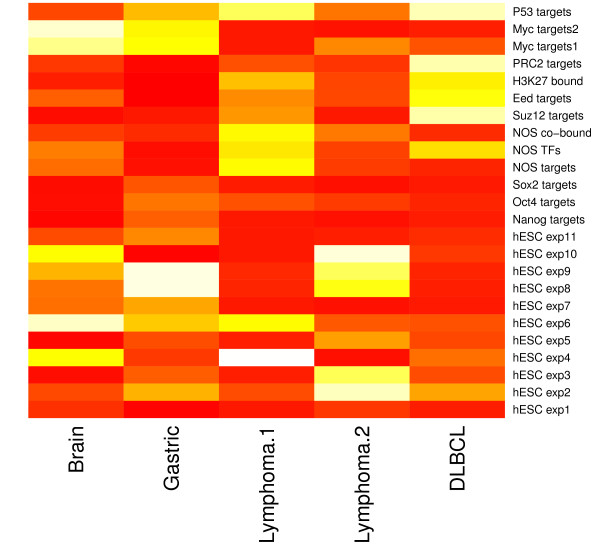
**Significance of overlap between hESC and tumor gene sets by survival analysis**. The detailed description of all the datasets is provided in Additional file 6.

We carried out significance analyses of the overlapping gene sets between each of the 72 DEGSs and each of the 24 hESC-associated gene sets based on the hypergeometric test. Three heatmaps of hypergeometric p-values are presented in Figure 1, Figure 2 and 3, which visualize the significance of the overlap between the hESC-associated gene sets and the DEGSs among normal vs. tumor, good prognosis vs. poor prognosis phenotypes classes, and survival analysis, respectively (the detailed description of all the datasets related to each figure is provided in Additional file 6). These figures show that the targets of three core hESC-associated TF OCT4, SOX2 and NANOG have significant overlaps with most of the DEGSs. Two gene sets targeted by MYC also shows significant overlaps with most of the DEGSs. These results suggest that key hESC-associated gene expression signatures have important implications in pathogenesis of cancer.

### Overlaps between hESCGESs pathways and tumor-associated pathways

In the total of 75 class comparison and survival analyses, we identified 68 groups of pathways significant at 0.05 threshold level. Among the 54 hESC-associated signal pathways signatures, 26 pathways appeared at least in eight different groups and the other 28 pathways didn't appear in any group. The most frequent identified pathway was the Cell Cycle pathway, which appeared for 57 times (84% occurrence rate), and the next one was the MAPK pathway which was identified for 50 times (74% occurrence rate). Table 7 lists all the 26 pathways and their occurrence frequencies in the 68 groups of pathways significant in the cancer datasets. These pathways have been proven to play important roles in both maintenance of hESC function and tumorigenesis.

**Table 7:** Twenty-six hESC-associated pathways frequently identified in tumors

Pathway	Frequency
Cell Cycle	57

MAPK	50

IGF	31

EGF/EGFR	30

ERK	29

SHH	28

AKT	26

RAS	25

NF-κB	22

Telomerase	22

p53	21

WNT	20

PRC2	19

ALK	16

NOTCH	15

ATM	14

VEGF	14

PDGF	13

ERBB2	11

JAK/STAT	11

PI3K	11

PTEN	11

TGFβ	11

MEK	10

STAT3	9

Glycolysis	8

Clearly, the Cell Cycle pathway plays an extremely important role in regulation of the self-renewal and pluripotency process of hESCs [55-59]. The undifferentiated hESCs have a short G1 phase, and therefore show rapid cell cycle characteristic relative to differentiated somatic cells. The unorthodox G1/S phase transition feature in the hESC cell cycle is associated with the deregulated proliferation and differentiation blockades of tumor cells [39,60-65]. The MAPK (Mitogen-Activated Protein Kinase) pathway regulates both the early embryonic development and the embryonic stem cell commitment from early steps of the process to mature differentiated cells [66]. The role of MAPK pathway in cancer is prominent as cancer can be perceived as a disease of communication between and within cells. The statistical significance analysis also shows that both the Cell Cycle pathway and MAPK pathway have important association with a majority of tumor types (see Additional file 7, Figure S1, Additional file 8, Figure S2 and Additional file 9, Figure S3).

The importance of IGF signaling pathway for maintenance of hESCs has been proven [67-70]. This signaling pathway appears to play a crucial role in cancer and can be of potential interest in cancer therapy [71-77]. The ERK pathway is active in the undifferentiation status of hESCs. Its activation is critical in maintenance of hESC self-renewal [78-81]. On the other hand, there has been accumulating evidence of ERK pathway (RAF-MEK-ERK signaling cascade) in oncogenesis to make it an attractive target for drug development [82].

Interestingly, almost all the widely-recognized hESC-associated pathways such as SHH, WNT, PRC2, Notch, PTEN and TGFβ have important linkage with cancer (see Table 7). The SHH (Sonic Hedgehog) signaling pathway is one of the key regulators of human embryonic development [83-87]. Activation of the pathway leads to an increased risk of the development of cancerous malignancies [87-94]. The WNT signaling pathway is a network of a number of proteins acting as a critical regulator of hESCs [43,56,59,69,79,84,85,95-103]. However, the deregulation of the pathway has been closely associated with cancer [83,86,90,94,103-114]. The PRC2 (Polycomb Repressive Complex 2) pathway is involved in control of the developmental regulators in hESCs [50,56,115-118]. The expression of PRC2 components is upregulated in various cancers such as melanoma, lymphoma, and breast and prostate cancer. The Notch signaling pathway plays a key role in the normal development of hESCs and many other cell types depending on the expression level and cellular context of the Notch receptors [84,85,101,119]. Its deregulation potentially contributes to cancer development in several different ways [111,120-126]. The PTEN (PhosphaTase and Tensin Homolog) acts as a tumor suppressor gene involved in regulation of the cell cycle, preventing cells from growing and dividing too rapidly. This pathway is also critical for stem cell maintenance [59,69,83]. The TGFβ (Transforming Growth Factor β) signaling pathway is of central importance to the self renewal of hESCs [43,59,69,79,84,85,96,98-102,115,127,128]. This signal pathway is involved in a wide range of cellular processes in both the adult organism and the developing embryo. It plays a role in both tumor suppression and tumor progression depending on cellular context [129-132].

Additional two important pathways involved in both hESCs function and tumorigenesis are p53 and telomerase pathways. They were identified for 21 and 22 times in our 68 class comparison or survival analysis (see Table 7). The p53 pathway can maintain the homeostasis of self-renewal and differentiation of hESCs [133-135]. Inactivation of this pathway in several cancer types may correlates with hESC-specific signatures [22,136,137]. Telomerase enzyme levels or activity has shown to be highly expressed in embryonic stem cells [79]. On the other hand, telomerase is reactivated and serves to maintain telomere length in most advanced cancers [34].

Taken together, the high overlap between hESCGESs pathways and tumor-associated pathways reveals that there exist common mechanisms underlying cancerous malignancies and "stemness" of hESCs.

### Overlaps between hESCGESs TFs and tumor-associated TFs

We identified 73 groups of targets of TFs significant at 0.05 threshold level. Among the 189 hESC-associated TF signatures, 42 TFs appeared at least in three different groups and the others didn't show in any group. The most frequently identified TF was MYC with 56% occurrence rate (41 occurrences), and the next one was MYB with 51% occurrence rate (37 occurrences). The complete 42 TFs accompanying with their occurrence frequencies are presented in Table 8.

**Table 8:** Forty-two hESC-associated TFs frequently identified in tumors

TF	Frequency
MYC	41

MYB	37

SP1	33

TP53	33

E2F4	33

TFAP2A	32

E2F1	32

JUN	30

SMAD1	27

TAL1	24

NFKB1	23

STAT3	23

MYBL2	22

ETS1	21

ETS2	20

POU2F1	20

POU2F2	18

STAT1	18

WT1	18

ETV4	18

HOXA9	17

SMAD3	17

E2F2	17

SP3	16

LEF1	14

NFKB2	14

POU5F1	13

PAX6	11

STAT4	11

SMAD4	10

GLI1	10

PAX3	9

PAX2	8

MYBL1	8

PAX5	7

SMAD2	7

GLI2	6

PAX8	6

STAT2	6

GLI3	3

POU3F2	3

E2F3	3

From Table 8, we can see a number of "stemness" TFs identified as informative in tumors. Evidently, MYC is one of the most important TFs in both hESCs and Cancer cells [22,23,44,48,49,52,56,116,138-140]. MYC represses differentiation and maintains the self-renewal of mouse and human pluripotent stem cells [138,141]. MYC regulatory networks may account for most of the transcriptional similarity between embryonic stem cells and cancer cells [139]. The statistical significance analysis also shows that MYC plays an important role in most of the tumor types analyzed (see Additional file 10, Figure S4, Additional file 11, Figure S5 and Additional file 12, Figure S6).

Another extremely important TF is POU5F1 (OCT4), which is necessary for induction of pluripotent stem cells from human somatic cells [23,24]. OCT4 constitutes the core transcriptional regulatory circuitry in hESCs in combination with SOX2 and NANOG essentially responsible for the early development and propagation of undifferentiated hESCs [43,44,52,56,58,59,79,84,97,116,117,119,142,143]. OCT4 expression appears to be important in maintaining the undifferentiated state of embryonal carcinoma [86,144], as well as in other cancers [27,145].

Our analysis results suggest that several families of hESC-associated TFs like MYB, E2F, PAX, SMAD, STAT, POU, SP and GLI, are related to cancer (Table 8). For example, three members of the TF family MYB: MYB, MYBL1 and MYBL2, appear to be closely associated with cancer (Table 8). In fact, a substantial number of studies have revealed that they had important roles in regulation of stem cell self-renewal and differentiation [146,147], and the development of cancer [148,149]. E2F plays a crucial role in control of the cell cycle progression and regulating the expression of genes required for G1/S transition [150], and therefore is important for stem cell self-renewal and differentiation. The members of the family E2F1, 2, 3 and E2F4 have been reported to be associated with cancer [151]. PAX plays an essential role in regulating cell proliferation and self-renewal, resistance to apoptosis, migration of embryonic precursor cells, and the coordination of specific differentiation programs during embryonic development [59], as well as the development of cancer [152]. SMAD regulates cell proliferation and differentiation by activating downstream TGFß gene transcription. Its members play important roles in hESC fate determination [98], and cancerous pathogenesis [153]. STAT regulates cell growth, survival and differentiation via activation by JAK (Janus kinase). This pathway is critical for regulation of stem cell self-renewal and differentiation [101]. Deregulation of this pathway is frequently observed in various tumor types [154]. POU mainly regulate the development of an organism, and are also involved in various cancers [155]. SP1 and SP3 are two members of the TF family SP (Specificity Protein) which binds GC-rich DNA sequences. Their roles in hESCs and cancer cells have been widely recognized [26]. GLI encompasses three members: GLI1, GLI2 and GLI3, all of which mediate the Hedgehog pathway and therefore are involved in hESC fate determination and cancerous pathogenesis [87].

In summary, the substantial overlap between the TFs involved in hESC fate determination and the TFs involved in cancerous pathogenesis suggests that hESCs and cancer cells may share essential regulatory mechanisms.

### Overlaps between hESCGESs miRNAs and Tumor-associated miRNAs

We identified 67 groups of miRNA targets significant at 0.05 threshold level. Among the 114 hESC-associated miRNA signatures, 102 miRNAs appeared at least in eight different groups and the other 12 miRNAs didn't show in any group. The most frequently identified miRNA was miR-29c, which occurred for 34 times (51% occurrence rate), and the next one was miR-200b which occurred for 30 times (45% occurrence rate). Table 9 lists 50 miRNAs whose occurrence frequencies are no less than 20. The complete 102 miRNAs accompanying with their occurrence frequencies are presented in Additional file 13, Table S5.

**Table 9:** Fifty hESC-associated miRNAs frequently identified in tumors

miRNA	Frequency
miR-29c	34

miR-200b	30

miR-19b	29

miR-29a	29

miR-29b	29

let-7a	28

miR-520f	28

miR-21	27

miR-302c	27

miR-302d	27

miR-494	27

miR-518b	27

miR-519c	27

miR-520a	27

miR-200c	26

miR-26a	26

miR-302a	26

miR-30d	26

miR-124a	25

miR-16	25

miR-19a	25

miR-302b	25

miR-374	25

miR-518c	25

miR-519b	25

miR-15a	24

miR-15b	24

miR-18a	24

miR-20b	24

miR-301	24

miR-520b	24

miR-520c	24

miR-130a	23

miR-18b	23

miR-369-3p	23

miR-520d	23

miR-520e	23

miR-103	22

miR-154	22

miR-20a	22

miR-525	22

miR-93	22

miR-17-5p	21

miR-302c*	21

miR-470	21

miR-515-5p	21

miR-517c	21

miR-106a	20

miR-146b	20

miR-96	20

Notably, there is a broad range of overlap between "stemness" miRNAs and oncogenic miRNAs. Most of the important "stemness" miRNAs are presented in Table 9 or Table S5. The miR-302 cluster miRNAs (miR-302a, miR-302a*, miR-302b, miR-302b*, miR-302c, miR-302c*, miR-302d) have been shown to regulate important cellular functions in hESCs, including cell proliferation and chromatin structure, and have been consistently reported to be overexpressed in hESCs [156]. All the seven members of this group appear in Table S5, and five of them are also presented in Table 9, indicative of their close linkage with cancer. Some literatures have reported the relatedness between miRNA-302 family and tumorigenecity [157-160]. Another group of miR-200 family miRNAs (miR-200a, miR-200b, miR-200c, miR-141 and miR-429) have been revealed to be hESC-specific, and upregulated in hESCs [156,161,162]. Three of them are presented in Table S5 and miR-200b and miR-200c are also listed in Table 9 with relatively high frequencies (30 and 26, respectively), strongly indicating their association with cancer. In fact, this miRNA family plays an important role in cancerous pathogenesis [163-165]. The miRNA-520 cluster on chromosome 19 was highly expressed in undifferentiated hESCs, and might be closely involved in hESC function [156,166]. Its eight members miRNA-520a-h show in Table S5 and six members miRNA-520a-f also show in Table 9, suggesting that the miRNA family has tight connection with cancer. Many studies have revealed the relatedness between its members and cancer [167-170]. The miR-518b, miR-518c, miR-519b and miR-519c have been consistently reported to be overexpressed in undifferentiated hESCs [156,166,171,172]. Our analysis outcomes suggest that they may be closely involved in the development of cancer (Table 9). This finding is supported by some studies [173,174]. In addition, the other miRNA families shown in Table 9 like miRNA-29, 19, 15, 20 and let-7 have been revealed to be involved in both hESC fate determination and cancerous pathogenesis [53,96,161,175].

The statistical significance analysis shows that some "stemness" miRNAs like miR-29 family member miR-29a, miR-29b and miR-29c are associated with a broad spectrum of tumor types (see Additional file 14, Figure S7, Additional file 15, Figure S8 and Additional file 16, Figure S9).

Taken together, a number of miRNAs play crucial roles in both hESC fate determination and tumorigenicity.

## Discussion

Although the evidence strongly supporting the CSC theory remains insufficient, and the fundamental experimental evidence for CSCs based on mouse xenograft models are controversial [21], the CSC model is attractive for it provides reasonable explanation of the development mechanisms underlying cancer, as well as a promise of improved cancer therapies. Therefore, any proof in favor of the CSC theory is valuable in the biology of cancer.

In this study, we provided an indirect evidence for the CSC theory using the computational biology approach. We found a strong linkage between hESCs and cancer cells by an examination of the similarity between the hESC-specific gene expression profiles and cancer-specific gene expression profiles. The hESC-specific gene expression signatures including genes, pathways, TFs and miRNAs were generally differentially expressed among normal vs. tumor phenotypes, or among cancer subtypes with distinct clinical outcomes. The genes important in regulation of hESC self-renewal and differentiation such as SOX2 and MYB, were also closely involved in tumorigenicity. The signal pathways such as the Cell Cycle, MAPK, SHH, WNT, PRC2, Notch, PTEN and TGFβ involved in the hESC fate determination were also strongly associated with cancer genesis, progression and prognosis. The typical hESC-specific TFs like OCT4 and c-Myc (also known as MYC), appeared to be important in control of the undifferentiated state of cancer cells. The miRNAs overexpressed in undifferentiated hESCs like miRNA-302, 200 and 520 cluster miRNAs, were closely involved in the development of cancer.

Generally speaking, the cell cycle regulation mechanism mostly underlies the commonality between hESCs and cancer cells. Differing from somatic cells, hESCs have an abbreviated G1 phase in cell cycle, which is critical for maintenance of hESC self-renewal and pluripotency. The abbreviated G1 phase is also largely responsible for the uncontrolled proliferation of tumor cells which escape from the programmed cell death during the G1 phase [62]. In fact, the hESC-associated signatures most frequently identified in tumors are mainly involved in regulation of cell cycle (see Table 6, Table 7, Table 8 and Table 9). Among them, the TF c-Myc is the core signature connecting hESCs with cancer cells. c-Myc binds genic and intergenic regions to regulate the expression of thousands of genes and noncoding RNAs throughout the genome [138]. c-Myc is involved in the cell cycle regulation by directly regulating cell cycle regulators [44,116,138], or regulating miRNAs which inhibit cell cycle regulators [96,138]. The role of c-Myc in linking hESCs with cancer has been recognized [138,139].

Here we identified differentially expressed genes at 0.05 significance level. A more stringent significance threshold of 0.001 would be more statistically reasonable if considering corrections of multiple hypotheses. Because the numbers of significant pathways, TFs and miRNAs identified by analyses of gene sets would be small for a majority of datasets if the significance threshold of 0.001 were used under which the number of differentially expressed genes were still often substantial, we selected the 0.05 significance level for all the differentially expressed analyses in order to keep consistency.

One limitation of this study was that the analyses were mainly based on the computational biology approach which needs experimental validation to corroborate these findings. In addition, some finer analyses such as grouping the overlaps of gene signatures between hESCs and tumors according to different tumor categories, separating the differentially expressed genes into the overexpressed and underexpressed genes etc., may contribute to a better understanding of the similarities between hESCs and tumor cells in gene expression profiles. Another limitation of this study was that we identified tumor-associated gene expression signatures based on whole tumor samples which might be derived from the majority of tumor cells, not necessarily from the minority of CSCs so that the overlapping signatures identified between hESCs and tumors might not be able to provide a strong support for the CSC model. If the tumor-associated gene expression signatures were identified by comparison between isolated CSCs versus non-CSCs fraction of the same tumor, the same results would be more reliable in support of the CSC model. These issues could be addressed in future research.

A further problem is the intertwined relationships between stem cell, cancer and ageing [176]. Cancer is actually an age-related disease as the incidence of cancer grows exponentially with ageing. Meanwhile, ageing is mostly caused by a decline in the replicative function of stem cell [177], and in turn aging has effects on the function of stem cell [178]. Thus, an in-depth investigation of the molecular mechanisms that connect stem cell, cancer and ageing will be necessary for postponing ageing and overcoming cancer.

## Conclusions

The present results revealed the close linkage between the hESC-specific gene expression profiles and cancer-specific gene expression profiles, and therefore offered an indirect support for the CSC theory. However, many interest issues remain to be addressed further.

## Availability of supporting data

The 51 human cancer gene expression datasets are available at the following website: http://linus.nci.nih.gov/~brb/DataArchive_New.html. All the other datasets supporting the results of this article are included within the article and its additional files.

## Competing interests

The author declares that he has no competing interests.

## Authors' contributions

XW designed and performed the research. XW wrote the manuscript.

## Supplementary Material

Additional file 1 **Table S1**. 24 hESC-associated gene sets. Click here for file

Additional file 2 **Table S2**. 189 hESC-associated transcription factors. Click here for file

Additional file 3 **Table S3**. 114 hESC-associated microRNAs. Click here for file

Additional file 4 **Supplementary references**. The list of references for the hESC-associated gene expression signatures identified and human tumor gene expression datasets used. Click here for file

Additional file 5 **Table S4**. 72 differentially expressed gene sets. Click here for file

Additional file 6 **Figure legends**. The list of legends for Figure 1, 2, 3 and S1-9. Click here for file

Additional file 7 **Figure S1. Significance of overlap between hESC and tumor pathways by normal vs. tumor class comparison**. The detailed description of all the datasets is provided in Additional file 6. Click here for file

Additional file 8 **Figure S2. Significance of overlap between hESC and tumor pathways by good vs. poor prognosis class comparison**. The detailed description of all the datasets is provided in Additional file 6. Click here for file

Additional file 9 **Figure S3. Significance of overlap between hESC and tumor pathways by survival analysis**. The detailed description of all the datasets is provided in Additional file 6. Click here for file

Additional file 10 **Figure S4. Significance of overlap between hESC and tumor TFs by normal vs. tumor class comparison**. The detailed description of all the datasets is provided in Additional file 6. Click here for file

Additional file 11 **Figure S5. Significance of overlap between hESC and tumor TFs by good vs. poor prognosis class comparison**. The detailed description of all the datasets is provided in Additional file 6. Click here for file

Additional file 12 **Figure S6. Significance of overlap between hESC and tumor TFs by survival analysis**. The detailed description of all the datasets is provided in Additional file 6. Click here for file

Additional file 13 **Table S5**. 102 miRNAs identified at least in eight different groups. Click here for file

Additional file 14 **Figure S7. Significance of overlap between hESC and tumor miRNAs by normal vs. tumor class comparison**. The detailed description of all the datasets is provided in Additional file 6. Click here for file

Additional file 15 **Figure S8. Significance of overlap between hESC and tumor miRNAs by good vs. poor prognosis class comparison**. The detailed description of all the datasets is provided in Additional file 6. Click here for file

Additional file 16 **Figure S9. Significance of overlap between hESC and tumor miRNAs by survival analysis**. The detailed description of all the datasets is provided in Additional file 6. Click here for file
