# Supplementary material for: Computational analysis of expression of human embryonic stem cell-associated signatures in tumors
Source: BMC Res Notes. 2011 Oct 31;4:471. doi: 10.1186/1756-0500-4-471 (PMC3217937; doi:10.1186/1756-0500-4-471)
Supplement: Additional file 4 — Supplementary references. The list of references for the hESC-associated gene expression signatures identified and human tumor gene expression datasets used. [file 1756-0500-4-471-S4.DOC]

**Table R1 - References for hESC-associated**  gene sets

| **Gene set** | **Reference** |
| --- | --- |
| **hESC exp1** | [1-2] |
| **hESC exp2** | [1-2] |
| **hESC exp3** | [1] |
| **hESC exp4** | [1] |
| **hESC exp5** | [2-9] |
| **hESC exp6** | [10] |
| **hESC exp7** | [8] |
| **hESC exp8** | [11] |
| **hESC exp9** | [11] |
| **hESC exp10** | [11] |
| **hESC exp11** | [12] |
| **Nanog targets** | [2-3] |
| **Oct4 targets** | [2-3] |
| **Sox2 targets** | [2-3] |
| **NOS targets** | [2-3] |
| **NOS TFs** | [2-3] |
| **NOS co-bound** | [3] |
| **Suz12 targets** | [2, 13] |
| **Eed targets** | [2, 13] |
| **H3K27 bound** | [2, 13] |
| **PRC2 targets** | [2, 13] |
| **Myc targets1** | [2, 14] |
| **Myc targets2** | [2, 15] |
| **P53 targets** | [16] |

**Table R2 - References for hESC-associated signal pathways**

| **Pathway** | **Reference** | |
| --- | --- | --- |
| Activin | [8, 17-24] | |
| AKT | [8, 24-25] | |
| ALK | [23, 26] | |
| ATM | [27-29] | |
| BMI1 | [30-35] | |
| BMP | [8, 21, 23-25, 36-38] | |
| Cell cycle | [4, 7-9, 21, 24-25, 39-45] | |
| EGF / EGFR | [46-48] | |
| ERBB2 | [48-49] | |
| ERK | [24, 41] | |
| FGF | [6-8, 11, 21-22, 24-25, 50-53] | |
| Glycolysis | [10, 54-57] | |
| Hippo | [54, 58] | |
| IGF | [25, 59-60] | |
| JAK / STAT | [8, 21, 51] | |
| c-KIT | [47, 54, 61] | |
| Lefty | [3, 6, 20-23, 38, 52, 62] | |
| LIF | [7, 24-25, 36-37, 63] | |
| MAPK | [24, 43, 64-65] | |
| MEK/ERK | [41, 53] | |
| NF-κB | [54, 66-69] | |
| NHEJ / HR | [70-71] | |
| Nodal | [8, 10, 20-24, 26, 51] | |
| Notch | [21, 50-51, 72] |  |
| p53 | [6-7, 16, 24-25, 41, 44, 54, 73-80] | |
| PRC2 | [6-7, 13, 30, 36] | |
| PDGF | [81-82] | |
| PI3K | [8, 24] | |
| PTEN | [8, 25, 35] | |
| RAS | [25, 45, 83] | |
| RTK | [51, 54, 67] | |
| Hedgehog (SHH) | [21, 31, 35, 50, 84] | |
| Smad | [6, 8, 20, 22-24, 26, 41] | |
| Stat3 | [3, 24, 36-37, 63, 85] | |
| Telomerase | [9, 24, 40, 79, 86-88] | |
| TGFβ | [3, 6, 8, 11, 20-25, 38, 41, 50-51, 54] | |
| VEGF | [89-91] | |
| WNT | [3, 7-8, 11, 20-21, 23-25, 38, 41, 50-52, 92-93] | |

**Table R3 - References for thirty hESC-associated TFs**

| **TF** | **Reference** |
| --- | --- |
| TP53 | [6-7, 24, 44, 75, 80] |
| MYC | [5, 9, 36] |
| GATA4 | [8, 13] |
| SMAD1 | [36-37] |
| ESRRB | [36-37] |
| SOX2 | [1, 3, 5, 36] |
| NANOG | [1, 3, 5, 8, 13, 36, 85] |
| KLF4 | [5, 36] [37] |
| MYB | [8] |
| MYCN | [36] |
| ZFX | [36-37, 94] |
| STAT3 | [3, 13, 36-37] |
| ZIC3 | [3, 13] |
| ZFP42 | [5, 7] |
| SALL4 | [7, 37] |
| REST | [3, 95] |
| TCF3 | [8, 11, 37] |
| HOXB1 | [3, 13] |
| HAND1 | [8, 13] |
| POU5F1 | [1, 3, 5, 8, 13, 36] |
| SRY | [7-8] |
| TBX5 | [8] |
| E2F4 | [3] |
| GATA6 | [10, 21, 52] |
| PAX6 | [3, 52] |
| TCF4 | [6, 51-52] |
| FOXD3 | [6, 52] |
| CNOT3 | [9, 94] |
| ZEB2 | [2, 13] |
| ESX1L | [3, 13] |

**Table R4 - References for twenty hESC-associated miRNAs**

| **miRNA ID** | **Reference** |
| --- | --- |
| miR-143 | [96] |
| miR-145 | [96-97] |
| miR-187 | [98] |
| miR-296 | [96, 99] |
| miR-301 | [99] |
| miR-21 | [4, 96, 100] |
| let-7a | [96, 99] |
| miR-371 | [96, 101-102] |
| miR-372 | [96, 100-103] |
| miR-373 | [100-102] |
| miR-367 | [96, 100, 103] |
| miR-302a | [4, 96, 98, 100, 102-103] |
| miR-302a* | [100, 103] |
| miR-302b | [4, 96, 98, 100, 102-103] |
| miR-302b* | [100, 102-103] |
| miR-302c | [96, 98, 100, 102-103] |
| miR-302c* | [100, 102] |
| miR-302d | [96, 98, 100, 102-103] |
| miR-200c | [96, 100, 103] |
| miR-222 | [4, 96, 100] |

**Table R5 - References for fifty-one human tumor gene expression datasets**

| **Tumor Type** | **# Datasets** | **Reference** |
| --- | --- | --- |
| Bladder Cancer | 1 | [104] |
| Brain Cancer | 6 | [105-110] |
| Breast Cancer | 5 | [111-115] |
| Colon Cancer | 1 | [116] |
| Cervical Cancer | 1 | [117] |
| Embryonal Cancer | 1 | [118] |
| Esophageal Cancer | 1 | [119] |
| Gastric Cancer | 2 | [120-121] |
| Head and Neck Cancer | 3 | [122-124] |
| Leukemia | 3 | [125-127] |
| Liver Cancer | 1 | [128] |
| Lung Cancer | 2 | [129-130] |
| Lymphoma | 4 | [131-134] |
| Medulloblastoma | 1 | [135] |
| Melanoma | 2 | [136-137] |
| Mesothelioma | 1 | [138] |
| Ovarian Cancer | 1 | [139] |
| Pancreatic Cancer | 1 | [140] |
| Prostate Cancer | 5 | [141-145] |
| Renal Cancer | 4 | [146-149] |
| Soft Tissue Sarcoma | 2 | [150-151] |
| Thyroid Cancer | 1 | [152] |
| Uterine Leiomyoma | 2 | [153-154] |

**Table R6 - References for all datasets with the algorithms performed**

| **Algorithms** | | **Datasets (references)** |
| --- | --- | --- |
| Class  comparison | normal  vs.  tumor | [104], [105], [108], [109], [117], [118], [119], [121], [120], [123], [130], [133], [136], [138], [140], [141], [142], [143], [144], [145], [146], [147], [151], [152], [153], [154] |
|  | good prognosis vs.  poor prognosis | [106], [107], [110], [111], [112], [113], [114], [115], [116], [119], [121], [122], [123], [124], [125], [126], [127], [128], [129], [132], [133], [134], [135], [137], [139], [141], [142], [145], [146], [149], [150], [154] |
| Survival analysis | | [110], [121], [131], [132], [133], [148] |

**Reference**

1. Assou S, Le Carrour T, Tondeur S, Strom S, Gabelle A, Marty S, Nadal L, Pantesco V, Reme T, Hugnot J-P *et al*: **A meta-analysis of human embryonic stem cells transcriptome integrated into a web-based expression atlas**. *Stem Cells* 2007, **25**(4):961-973.

2. Ben-Porath I, Thomson MW, Carey VJ, Ge R, Bell GW, Regev A, Weinberg RA: **An embryonic stem cell-like gene expression signature in poorly differentiated aggressive human tumors**. *Nature genetics* 2008, **40**(5):499-507.

3. Boyer LA, Lee TI, Cole MF, Johnstone SE, Levine SS, Zucker JP, Guenther MG, Kumar RM, Murray HL, Jenner RG *et al*: **Core transcriptional regulatory circuitry in human embryonic stem cells**. *Cell* 2005, **122**(6):947-956.

4. Morin RD, O'Connor MD, Griffith M, Kuchenbauer F, Delaney A, Prabhu A-L, Zhao Y, McDonald H, Zeng T, Hirst M *et al*: **Application of massively parallel sequencing to microRNA profiling and discovery in human embryonic stem cells**. *Genome Res* 2008, **18**(4):610-621.

5. Kim J, Chu J, Shen X, Wang J, Orkin SH: **An extended transcriptional network for pluripotency of embryonic stem cells**. *Cell* 2008, **132**(6):1049-1061.

6. Wang Y, Mah N, Prigione A, Wolfrum K, Andrade-Navarro MA, Adjaye J: **A transcriptional roadmap to the induction of pluripotency in somatic cells**. *Stem Cell Rev* 2010, **6**(2):282-296.

7. Kashyap V, Rezende NC, Scotland KB, Shaffer SM, Persson JL, Gudas LJ, Mongan NP: **Regulation of stem cell pluripotency and differentiation involves a mutual regulatory circuit of the NANOG, OCT4, and SOX2 pluripotency transcription factors with polycomb repressive complexes and stem cell microRNAs**. *Stem Cells Dev* 2009, **18**(7):1093-1108.

8. Sun Y, Li H, Liu Y, Mattson MP, Rao MS, Zhan M: **Evolutionarily conserved transcriptional co-expression guiding embryonic stem cell differentiation**. *PLoS One* 2008, **3**(10):e3406.

9. Smith K, Dalton S: **Myc transcription factors: key regulators behind establishment and maintenance of pluripotency**. *Regen Med* 2010, **5**(6):947-959.

10. Adewumi O, Aflatoonian B, Ahrlund-Richter L, Amit M, Andrews PW, Beighton G, Bello PA, Benvenisty N, Berry LS, Bevan S *et al*: **Characterization of human embryonic stem cell lines by the International Stem Cell Initiative**. *Nat Biotechnol* 2007, **25**(7):803-816.

11. Richards M, Tan S-P, Tan J-H, Chan W-K, Bongso A: **The transcriptome profile of human embryonic stem cells as defined by SAGE**. *Stem Cells* 2004, **22**(1):51-64.

12. Sperger JM, Chen X, Draper JS, Antosiewicz JE, Chon CH, Jones SB, Brooks JD, Andrews PW, Brown PO, Thomson JA: **Gene expression patterns in human embryonic stem cells and human pluripotent germ cell tumors**. *Proceedings of the National Academy of Sciences of the United States of America* 2003, **100**(23):13350-13355.

13. Lee TI, Jenner RG, Boyer LA, Guenther MG, Levine SS, Kumar RM, Chevalier B, Johnstone SE, Cole MF, Isono K-i *et al*: **Control of developmental regulators by Polycomb in human embryonic stem cells**. *Cell* 2006, **125**(2):301-313.

14. Fernandez PC, Frank SR, Wang L, Schroeder M, Liu S, Greene J, Cocito A, Amati B: **Genomic targets of the human c-Myc protein**. *Genes Dev* 2003, **17**(9):1115-1129.

15. Li Z, Van Calcar S, Qu C, Cavenee WK, Zhang MQ, Ren B: **A global transcriptional regulatory role for c-Myc in Burkitt's lymphoma cells**. *Proc Natl Acad Sci U S A* 2003, **100**(14):8164-8169.

16. Hong H, Takahashi K, Ichisaka T, Aoi T, Kanagawa O, Nakagawa M, Okita K, Yamanaka S: **Suppression of induced pluripotent stem cell generation by the p53-p21 pathway**. *Nature* 2009, **460**(7259):1132-1135.

17. Tsuchida K, Nakatani M, Hitachi K, Uezumi A, Sunada Y, Ageta H, Inokuchi K: **Activin signaling as an emerging target for therapeutic interventions**. *Cell Commun Signal* 2009, **7**:15.

18. Xiao L, Yuan X, Sharkis SJ: **Activin A maintains self-renewal and regulates fibroblast growth factor, Wnt, and bone morphogenic protein pathways in human embryonic stem cells**. *Stem Cells* 2006, **24**(6):1476-1486.

19. Xu R-H, Sampsell-Barron TL, Gu F, Root S, Peck RM, Pan G, Yu J, Antosiewicz-Bourget J, Tian S, Stewart R *et al*: **NANOG is a direct target of TGFbeta/activin-mediated SMAD signaling in human ESCs**. *Cell Stem Cell* 2008, **3**(2):196-206.

20. James D, Levine AJ, Besser D, Hemmati-Brivanlou A: **TGFbeta/activin/nodal signaling is necessary for the maintenance of pluripotency in human embryonic stem cells**. *Development* 2005, **132**(6):1273-1282.

21. Babaie Y, Herwig R, Greber B, Brink TC, Wruck W, Groth D, Lehrach H, Burdon T, Adjaye J: **Analysis of Oct4-dependent transcriptional networks regulating self-renewal and pluripotency in human embryonic stem cells**. *Stem Cells* 2007, **25**(2):500-510.

22. Vallier L, Alexander M, Pedersen RA: **Activin/Nodal and FGF pathways cooperate to maintain pluripotency of human embryonic stem cells**. *J Cell Sci* 2005, **118**(Pt 19):4495-4509.

23. Besser D: **Expression of nodal, lefty-a, and lefty-B in undifferentiated human embryonic stem cells requires activation of Smad2/3**. *The Journal of biological chemistry* 2004, **279**(43):45076-45084.

24. Liu N, Lu M, Tian X, Han Z: **Molecular mechanisms involved in self-renewal and pluripotency of embryonic stem cells**. *J Cell Physiol* 2007, **211**(2):279-286.

25. Brandenberger R, Khrebtukova I, Thies RS, Miura T, Jingli C, Puri R, Vasicek T, Lebkowski J, Rao M: **MPSS profiling of human embryonic stem cells**. *BMC Dev Biol* 2004, **4**:10.

26. Hendrix MJC, Seftor EA, Seftor REB, Kasemeier-Kulesa J, Kulesa PM, Postovit L-M: **Reprogramming metastatic tumour cells with embryonic microenvironments**. *Nat Rev Cancer* 2007, **7**(4):246-255.

27. Wong K-K, Maser RS, Bachoo RM, Menon J, Carrasco DR, Gu Y, Alt FW, DePinho RA: **Telomere dysfunction and Atm deficiency compromises organ homeostasis and accelerates ageing**. *Nature* 2003, **421**(6923):643-648.

28. Ito K, Hirao A, Arai F, Matsuoka S, Takubo K, Hamaguchi I, Nomiyama K, Hosokawa K, Sakurada K, Nakagata N *et al*: **Regulation of oxidative stress by ATM is required for self-renewal of haematopoietic stem cells**. *Nature* 2004, **431**(7011):997-1002.

29. Moore KA, Lemischka IR: **Stem cells and their niches**. *Science* 2006, **311**(5769):1880-1885.

30. Glinsky GV: **"Stemness" genomics law governs clinical behavior of human cancer: implications for decision making in disease management**. *Journal of clinical oncology : official journal of the American Society of Clinical Oncology* 2008, **26**(17):2846-2853.

31. Liu S, Dontu G, Mantle ID, Patel S, Ahn N-s, Jackson KW, Suri P, Wicha MS: **Hedgehog signaling and Bmi-1 regulate self-renewal of normal and malignant human mammary stem cells**. *Cancer research* 2006, **66**(12):6063-6071.

32. Park I-k, Qian D, Kiel M, Becker MW, Pihalja M, Weissman IL, Morrison SJ, Clarke MF: **Bmi-1 is required for maintenance of adult self-renewing haematopoietic stem cells**. *Nature* 2003, **423**(6937):302-305.

33. Lessard J, Sauvageau G: **Bmi-1 determines the proliferative capacity of normal and leukaemic stem cells**. *Nature* 2003, **423**(6937):255-260.

34. Molofsky AV, Pardal R, Iwashita T, Park I-K, Clarke MF, Morrison SJ: **Bmi-1 dependence distinguishes neural stem cell self-renewal from progenitor proliferation**. *Nature* 2003, **425**(6961):962-967.

35. Pardal R, Clarke MF, Morrison SJ: **Applying the principles of stem-cell biology to cancer**. *Nat Rev Cancer* 2003, **3**(12):895-902.

36. Chen X, Xu H, Yuan P, Fang F, Huss M, Vega VB, Wong E, Orlov YL, Zhang W, Jiang J *et al*: **Integration of external signaling pathways with the core transcriptional network in embryonic stem cells**. *Cell* 2008, **133**(6):1106-1117.

37. Young RA: **Control of the embryonic stem cell state**. *Cell* 2011, **144**(6):940-954.

38. Rao RR, Stice SL: **Gene expression profiling of embryonic stem cells leads to greater understanding of pluripotency and early developmental events**. *Biol Reprod* 2004, **71**(6):1772-1778.

39. Levi E, Misra S, Du J, Patel BB, Majumdar APN: **Combination of aging and dimethylhydrazine treatment causes an increase in cancer-stem cell population of rat colonic crypts**. *Biochem Biophys Res Commun* 2009, **385**(3):430-433.

40. Ben-David U, Benvenisty N: **The tumorigenicity of human embryonic and induced pluripotent stem cells**. *Nat Rev Cancer* 2011, **11**(4):268-277.

41. Mallanna SK, Rizzino A: **Emerging roles of microRNAs in the control of embryonic stem cells and the generation of induced pluripotent stem cells**. *Dev Biol* 2010, **344**(1):16-25.

42. Card DAG, Hebbar PB, Li L, Trotter KW, Komatsu Y, Mishina Y, Archer TK: **Oct4/Sox2-regulated miR-302 targets cyclin D1 in human embryonic stem cells**. *Mol Cell Biol* 2008, **28**(20):6426-6438.

43. Jung M, Peterson H, Chavez L, Kahlem P, Lehrach H, Vilo J, Adjaye J: **A data integration approach to mapping OCT4 gene regulatory networks operative in embryonic stem cells and embryonal carcinoma cells**. *PLoS One* 2010, **5**(5):e10709.

44. Field M, Alvarez A, Bushnev S, Sugaya K: **Embryonic stem cell markers distinguishing cancer stem cells from normal human neuronal stem cell populations in malignant glioma patients**. *Clin Neurosurg* 2010, **57**:151-159.

45. Burdon T, Smith A, Savatier P: **Signalling, cell cycle and pluripotency in embryonic stem cells**. *Trends Cell Biol* 2002, **12**(9):432-438.

46. Aguirre A, Rubio ME, Gallo V: **Notch and EGFR pathway interaction regulates neural stem cell number and self-renewal**. *Nature* 2010, **467**(7313):323-327.

47. Mimeault M, Hauke R, Mehta PP, Batra SK: **Recent advances in cancer stem/progenitor cell research: therapeutic implications for overcoming resistance to the most aggressive cancers**. *J Cell Mol Med* 2007, **11**(5):981-1011.

48. Kornblum HI, Yanni DS, Easterday MC, Seroogy KB: **Expression of the EGF receptor family members ErbB2, ErbB3, and ErbB4 in germinal zones of the developing brain and in neurosphere cultures containing CNS stem cells**. *Dev Neurosci* 2000, **22**(1-2):16-24.

49. Wang L, Schulz TC, Sherrer ES, Dauphin DS, Shin S, Nelson AM, Ware CB, Zhan M, Song C-Z, Chen X *et al*: **Self-renewal of human embryonic stem cells requires insulin-like growth factor-1 receptor and ERBB2 receptor signaling**. *Blood* 2007, **110**(12):4111-4119.

50. Katoh M, Katoh M: **WNT signaling pathway and stem cell signaling network**. *Clinical cancer research : an official journal of the American Association for Cancer Research* 2007, **13**(14):4042-4045.

51. Rho J-Y, Yu K, Han J-S, Chae J-I, Koo D-B, Yoon H-S, Moon S-Y, Lee K-K, Han Y-M: **Transcriptional profiling of the developmentally important signalling pathways in human embryonic stem cells**. *Hum Reprod* 2006, **21**(2):405-412.

52. Chavez L, Bais AS, Vingron M, Lehrach H, Adjaye J, Herwig R: **In silico identification of a core regulatory network of OCT4 in human embryonic stem cells using an integrated approach**. *BMC Genomics* 2009, **10**:314.

53. Song Z, Yue W, Wei B, Wang N, Li T, Guan L, Shi S, Zeng Q, Pei X, Chen L: **Sonic hedgehog pathway is essential for maintenance of cancer stem-like cells in human gastric cancer**. *PLoS One* 2011, **6**(3):e17687.

54. Zoller M: **CD44: can a cancer-initiating cell profit from an abundantly expressed molecule?** *Nat Rev Cancer* 2011, **11**(4):254-267.

55. Chung S, Arrell DK, Faustino RS, Terzic A, Dzeja PP: **Glycolytic network restructuring integral to the energetics of embryonic stem cell cardiac differentiation**. *J Mol Cell Cardiol* 2010, **48**(4):725-734.

56. Kondoh H, Lleonart ME, Bernard D, Gil J: **Protection from oxidative stress by enhanced glycolysis; a possible mechanism of cellular immortalization**. *Histol Histopathol* 2007, **22**(1):85-90.

57. Funes JM, Quintero M, Henderson S, Martinez D, Qureshi U, Westwood C, Clements MO, Bourboulia D, Pedley RB, Moncada S *et al*: **Transformation of human mesenchymal stem cells increases their dependency on oxidative phosphorylation for energy production**. *Proc Natl Acad Sci U S A* 2007, **104**(15):6223-6228.

58. Halder G, Johnson RL: **Hippo signaling: growth control and beyond**. *Development* 2011, **138**(1):9-22.

59. Celil AB, Campbell PG: **BMP-2 and insulin-like growth factor-I mediate Osterix (Osx) expression in human mesenchymal stem cells via the MAPK and protein kinase D signaling pathways**. *J Biol Chem* 2005, **280**(36):31353-31359.

60. Rafalski VA, Brunet A: **Energy metabolism in adult neural stem cell fate**. *Prog Neurobiol* 2011, **93**(2):182-203.

61. Wandzioch E, Edling CE, Palmer RH, Carlsson L, Hallberg B: **Activation of the MAP kinase pathway by c-Kit is PI-3 kinase dependent in hematopoietic progenitor/stem cell lines**. *Blood* 2004, **104**(1):51-57.

62. Dvash T, Sharon N, Yanuka O, Benvenisty N: **Molecular analysis of LEFTY-expressing cells in early human embryoid bodies**. *Stem Cells* 2007, **25**(2):465-472.

63. Kidder BL, Yang J, Palmer S: **Stat3 and c-Myc genome-wide promoter occupancy in embryonic stem cells**. *PLoS One* 2008, **3**(12):e3932.

64. Binetruy B, Heasley L, Bost F, Caron L, Aouadi M: **Concise review: regulation of embryonic stem cell lineage commitment by mitogen-activated protein kinases**. *Stem Cells* 2007, **25**(5):1090-1095.

65. Campos LS, Leone DP, Relvas JB, Brakebusch C, Fassler R, Suter U, ffrench-Constant C: **Beta1 integrins activate a MAPK signalling pathway in neural stem cells that contributes to their maintenance**. *Development* 2004, **131**(14):3433-3444.

66. Dreesen O, Brivanlou AH: **Signaling pathways in cancer and embryonic stem cells**. *Stem Cell Rev* 2007, **3**(1):7-17.

67. Polyak K, Weinberg RA: **Transitions between epithelial and mesenchymal states: acquisition of malignant and stem cell traits**. *Nat Rev Cancer* 2009, **9**(4):265-273.

68. Memet S: **NF-kappaB functions in the nervous system: from development to disease**. *Biochem Pharmacol* 2006, **72**(9):1180-1195.

69. Novotny NM, Markel TA, Crisostomo PR, Meldrum DR: **Differential IL-6 and VEGF secretion in adult and neonatal mesenchymal stem cells: role of NFkB**. *Cytokine* 2008, **43**(2):215-219.

70. Park Y, Gerson SL: **DNA repair defects in stem cell function and aging**. *Annu Rev Med* 2005, **56**:495-508.

71. Serrano L, Liang L, Chang Y, Deng L, Maulion C, Nguyen S, Tischfield JA: **Homologous recombination conserves DNA sequence integrity throughout the cell cycle in embryonic stem cells**. *Stem Cells Dev* 2011, **20**(2):363-374.

72. Chambers I, Tomlinson SR: **The transcriptional foundation of pluripotency**. *Development* 2009, **136**(14):2311-2322.

73. Song H, Chung S-K, Xu Y: **Modeling disease in human ESCs using an efficient BAC-based homologous recombination system**. *Cell Stem Cell* 2010, **6**(1):80-89.

74. Krizhanovsky V, Lowe SW: **Stem cells: The promises and perils of p53**. *Nature* 2009, **460**(7259):1085-1086.

75. Kawamura T, Suzuki J, Wang YV, Menendez S, Morera LB, Raya A, Wahl GM, Belmonte JCI: **Linking the p53 tumour suppressor pathway to somatic cell reprogramming**. *Nature* 2009, **460**(7259):1140-1144.

76. Jerry DJ, Tao L, Yan H: **Regulation of cancer stem cells by p53**. *Breast Cancer Res* 2008, **10**(4):304.

77. Aparicio S, Eaves CJ: **p53: a new kingpin in the stem cell arena**. *Cell* 2009, **138**(6):1060-1062.

78. Cicalese A, Bonizzi G, Pasi CE, Faretta M, Ronzoni S, Giulini B, Brisken C, Minucci S, Di Fiore PP, Pelicci PG: **The tumor suppressor p53 regulates polarity of self-renewing divisions in mammary stem cells**. *Cell* 2009, **138**(6):1083-1095.

79. Menendez S, Camus S, Izpisua Belmonte JC: **p53: guardian of reprogramming**. *Cell cycle (Georgetown, Tex )* 2010, **9**(19):3887-3891.

80. Mizuno H, Spike BT, Wahl GM, Levine AJ: **Inactivation of p53 in breast cancers correlates with stem cell transcriptional signatures**. *Proc Natl Acad Sci U S A* 2010, **107**(52):22745-22750.

81. Pebay A, Wong RCB, Pitson SM, Wolvetang EJ, Peh GSL, Filipczyk A, Koh KLL, Tellis I, Nguyen LTV, Pera MF: **Essential roles of sphingosine-1-phosphate and platelet-derived growth factor in the maintenance of human embryonic stem cells**. *Stem Cells* 2005, **23**(10):1541-1548.

82. Jackson EL, Garcia-Verdugo JM, Gil-Perotin S, Roy M, Quinones-Hinojosa A, VandenBerg S, Alvarez-Buylla A: **PDGFR alpha-positive B cells are neural stem cells in the adult SVZ that form glioma-like growths in response to increased PDGF signaling**. *Neuron* 2006, **51**(2):187-199.

83. Yang W, Klaman LD, Chen B, Araki T, Harada H, Thomas SM, George EL, Neel BG: **An Shp2/SFK/Ras/Erk signaling pathway controls trophoblast stem cell survival**. *Dev Cell* 2006, **10**(3):317-327.

84. Sell S: **Stem cell origin of cancer and differentiation therapy**. *Crit Rev Oncol Hematol* 2004, **51**(1):1-28.

85. Chambers I, Colby D, Robertson M, Nichols J, Lee S, Tweedie S, Smith A: **Functional expression cloning of Nanog, a pluripotency sustaining factor in embryonic stem cells**. *Cell* 2003, **113**(5):643-655.

86. Kim J, Woo AJ, Chu J, Snow JW, Fujiwara Y, Kim CG, Cantor AB, Orkin SH: **A Myc network accounts for similarities between embryonic stem and cancer cell transcription programs**. *Cell* 2010, **143**(2):313-324.

87. Yu J, Vodyanik MA, Smuga-Otto K, Antosiewicz-Bourget J, Frane JL, Tian S, Nie J, Jonsdottir GA, Ruotti V, Stewart R *et al*: **Induced pluripotent stem cell lines derived from human somatic cells**. *Science (New York, N Y )* 2007, **318**(5858):1917-1920.

88. Marion RM, Strati K, Li H, Tejera A, Schoeftner S, Ortega S, Serrano M, Blasco MA: **Telomeres acquire embryonic stem cell characteristics in induced pluripotent stem cells**. *Cell Stem Cell* 2009, **4**(2):141-154.

89. Gerber H-P, Malik AK, Solar GP, Sherman D, Liang XH, Meng G, Hong K, Marsters JC, Ferrara N: **VEGF regulates haematopoietic stem cell survival by an internal autocrine loop mechanism**. *Nature* 2002, **417**(6892):954-958.

90. Orkin SH, Zon LI: **Hematopoiesis: an evolving paradigm for stem cell biology**. *Cell* 2008, **132**(4):631-644.

91. Sun J, Zhou W, Ma D, Yang Y: **Endothelial cells promote neural stem cell proliferation and differentiation associated with VEGF activated Notch and Pten signaling**. *Dev Dyn* 2010, **239**(9):2345-2353.

92. Sato N, Meijer L, Skaltsounis L, Greengard P, Brivanlou AH: **Maintenance of pluripotency in human and mouse embryonic stem cells through activation of Wnt signaling by a pharmacological GSK-3-specific inhibitor**. *Nat Med* 2004, **10**(1):55-63.

93. Reya T, Clevers H: **Wnt signalling in stem cells and cancer**. *Nature* 2005, **434**(7035):843-850.

94. Hu G, Kim J, Xu Q, Leng Y, Orkin SH, Elledge SJ: **A genome-wide RNAi screen identifies a new transcriptional module required for self-renewal**. *Genes Dev* 2009, **23**(7):837-848.

95. Singh SK, Kagalwala MN, Parker-Thornburg J, Adams H, Majumder S: **REST maintains self-renewal and pluripotency of embryonic stem cells**. *Nature* 2008, **453**(7192):223-227.

96. Lakshmipathy U, Love B, Goff LA, Jornsten R, Graichen R, Hart RP, Chesnut JD: **MicroRNA expression pattern of undifferentiated and differentiated human embryonic stem cells**. *Stem Cells Dev* 2007, **16**(6):1003-1016.

97. Xu N, Papagiannakopoulos T, Pan G, Thomson JA, Kosik KS: **MicroRNA-145 regulates OCT4, SOX2, and KLF4 and represses pluripotency in human embryonic stem cells**. *Cell* 2009, **137**(4):647-658.

98. Bar M, Wyman SK, Fritz BR, Qi J, Garg KS, Parkin RK, Kroh EM, Bendoraite A, Mitchell PS, Nelson AM *et al*: **MicroRNA discovery and profiling in human embryonic stem cells by deep sequencing of small RNA libraries**. *Stem Cells* 2008, **26**(10):2496-2505.

99. Gangaraju VK, Lin H: **MicroRNAs: key regulators of stem cells**. *Nat Rev Mol Cell Biol* 2009, **10**(2):116-125.

100. Suh M-R, Lee Y, Kim JY, Kim S-K, Moon S-H, Lee JY, Cha K-Y, Chung HM, Yoon HS, Moon SY *et al*: **Human embryonic stem cells express a unique set of microRNAs**. *Dev Biol* 2004, **270**(2):488-498.

101. Laurent LC, Chen J, Ulitsky I, Mueller F-J, Lu C, Shamir R, Fan J-B, Loring JF: **Comprehensive microRNA profiling reveals a unique human embryonic stem cell signature dominated by a single seed sequence**. *Stem Cells* 2008, **26**(6):1506-1516.

102. Barroso-delJesus A, Romero-Lopez C, Lucena-Aguilar G, Melen GJ, Sanchez L, Ligero G, Berzal-Herranz A, Menendez P: **Embryonic stem cell-specific miR302-367 cluster: human gene structure and functional characterization of its core promoter**. *Mol Cell Biol* 2008, **28**(21):6609-6619.

103. Ren J, Jin P, Wang E, Marincola FM, Stroncek DF: **MicroRNA and gene expression patterns in the differentiation of human embryonic stem cells**. *J Transl Med* 2009, **7**:20.

104. Dyrskjot L, Thykjaer T, Kruhoffer M, Jensen JL, Marcussen N, Hamilton-Dutoit S, Wolf H, Orntoft TF: **Identifying distinct classes of bladder carcinoma using microarrays**. *Nat Genet* 2003, **33**(1):90-96.

105. Bredel M, Bredel C, Juric D, Harsh GR, Vogel H, Recht LD, Sikic BI: **Functional network analysis reveals extended gliomagenesis pathway maps and three novel MYC-interacting genes in human gliomas**. *Cancer Res* 2005, **65**(19):8679-8689.

106. Bredel M, Bredel C, Juric D, Duran GE, Yu RX, Harsh GR, Vogel H, Recht LD, Scheck AC, Sikic BI: **Tumor necrosis factor-alpha-induced protein 3 as a putative regulator of nuclear factor-kappaB-mediated resistance to O6-alkylating agents in human glioblastomas**. *J Clin Oncol* 2006, **24**(2):274-287.

107. Dong S, Nutt CL, Betensky RA, Stemmer-Rachamimov AO, Denko NC, Ligon KL, Rowitch DH, Louis DN: **Histology-based expression profiling yields novel prognostic markers in human glioblastoma**. *J Neuropathol Exp Neurol* 2005, **64**(11):948-955.

108. Sun L, Hui A-M, Su Q, Vortmeyer A, Kotliarov Y, Pastorino S, Passaniti A, Menon J, Walling J, Bailey R *et al*: **Neuronal and glioma-derived stem cell factor induces angiogenesis within the brain**. *Cancer cell* 2006, **9**(4):287-300.

109. Liang Y, Diehn M, Watson N, Bollen AW, Aldape KD, Nicholas MK, Lamborn KR, Berger MS, Botstein D, Brown PO *et al*: **Gene expression profiling reveals molecularly and clinically distinct subtypes of glioblastoma multiforme**. *Proceedings of the National Academy of Sciences of the United States of America* 2005, **102**(16):5814-5819.

110. Pomeroy SL, Tamayo P, Gaasenbeek M, Sturla LM, Angelo M, McLaughlin ME, Kim JYH, Goumnerova LC, Black PM, Lau C *et al*: **Prediction of central nervous system embryonal tumour outcome based on gene expression**. *Nature* 2002, **415**(6870):436-442.

111. Chang JC, Wooten EC, Tsimelzon A, Hilsenbeck SG, Gutierrez MC, Elledge R, Mohsin S, Osborne CK, Chamness GC, Allred DC *et al*: **Gene expression profiling for the prediction of therapeutic response to docetaxel in patients with breast cancer**. *Lancet* 2003, **362**(9381):362-369.

112. Ma X-J, Wang Z, Ryan PD, Isakoff SJ, Barmettler A, Fuller A, Muir B, Mohapatra G, Salunga R, Tuggle JT *et al*: **A two-gene expression ratio predicts clinical outcome in breast cancer patients treated with tamoxifen**. *Cancer cell* 2004, **5**(6):607-616.

113. Sorlie T, Tibshirani R, Parker J, Hastie T, Marron JS, Nobel A, Deng S, Johnsen H, Pesich R, Geisler S *et al*: **Repeated observation of breast tumor subtypes in independent gene expression data sets**. *Proceedings of the National Academy of Sciences of the United States of America* 2003, **100**(14):8418-8423.

114. Sotiriou C, Neo S-Y, McShane LM, Korn EL, Long PM, Jazaeri A, Martiat P, Fox SB, Harris AL, Liu ET: **Breast cancer classification and prognosis based on gene expression profiles from a population-based study**. *Proceedings of the National Academy of Sciences of the United States of America* 2003, **100**(18):10393-10398.

115. van 't Veer LJ, Dai H, van de Vijver MJ, He YD, Hart AAM, Mao M, Peterse HL, van der Kooy K, Marton MJ, Witteveen AT *et al*: **Gene expression profiling predicts clinical outcome of breast cancer**. *Nature* 2002, **415**(6871):530-536.

116. Koinuma K, Yamashita Y, Liu W, Hatanaka H, Kurashina K, Wada T, Takada S, Kaneda R, Choi YL, Fujiwara SI *et al*: **Epigenetic silencing of AXIN2 in colorectal carcinoma with microsatellite instability**. *Oncogene* 2006, **25**(1):139-146.

117. Wong YF, Selvanayagam ZE, Wei N, Porter J, Vittal R, Hu R, Lin Y, Liao J, Shih JW, Cheung TH *et al*: **Expression genomics of cervical cancer: molecular classification and prediction of radiotherapy response by DNA microarray**. *Clin Cancer Res* 2003, **9**(15):5486-5492.

118. Skotheim RI, Lind GE, Monni O, Nesland JM, Abeler VM, Fossa SD, Duale N, Brunborg G, Kallioniemi O, Andrews PW *et al*: **Differentiation of human embryonal carcinomas in vitro and in vivo reveals expression profiles relevant to normal development**. *Cancer research* 2005, **65**(13):5588-5598.

119. Kimchi ET, Posner MC, Park JO, Darga TE, Kocherginsky M, Karrison T, Hart J, Smith KD, Mezhir JJ, Weichselbaum RR *et al*: **Progression of Barrett's metaplasia to adenocarcinoma is associated with the suppression of the transcriptional programs of epidermal differentiation**. *Cancer Res* 2005, **65**(8):3146-3154.

120. Hippo Y, Taniguchi H, Tsutsumi S, Machida N, Chong J-M, Fukayama M, Kodama T, Aburatani H: **Global gene expression analysis of gastric cancer by oligonucleotide microarrays**. *Cancer Res* 2002, **62**(1):233-240.

121. Chen X, Leung SY, Yuen ST, Chu K-M, Ji J, Li R, Chan ASY, Law S, Troyanskaya OG, Wong J *et al*: **Variation in gene expression patterns in human gastric cancers**. *Mol Biol Cell* 2003, **14**(8):3208-3215.

122. Chung CH, Parker JS, Karaca G, Wu J, Funkhouser WK, Moore D, Butterfoss D, Xiang D, Zanation A, Yin X *et al*: **Molecular classification of head and neck squamous cell carcinomas using patterns of gene expression**. *Cancer cell* 2004, **5**(5):489-500.

123. Cromer A, Carles A, Millon R, Ganguli G, Chalmel F, Lemaire F, Young J, Dembele D, Thibault C, Muller D *et al*: **Identification of genes associated with tumorigenesis and metastatic potential of hypopharyngeal cancer by microarray analysis**. *Oncogene* 2004, **23**(14):2484-2498.

124. O'Donnell RK, Kupferman M, Wei SJ, Singhal S, Weber R, O'Malley B, Cheng Y, Putt M, Feldman M, Ziober B *et al*: **Gene expression signature predicts lymphatic metastasis in squamous cell carcinoma of the oral cavity**. *Oncogene* 2005, **24**(7):1244-1251.

125. Crossman LC, Mori M, Hsieh Y-C, Lange T, Paschka P, Harrington CA, Krohn K, Niederwieser DW, Hehlmann R, Hochhaus A *et al*: **In chronic myeloid leukemia white cells from cytogenetic responders and non-responders to imatinib have very similar gene expression signatures**. *Haematologica* 2005, **90**(4):459-464.

126. Falt S, Merup M, Gahrton G, Lambert B, Wennborg A: **Identification of progression markers in B-CLL by gene expression profiling**. *Exp Hematol* 2005, **33**(8):883-893.

127. Yagi T, Morimoto A, Eguchi M, Hibi S, Sako M, Ishii E, Mizutani S, Imashuku S, Ohki M, Ichikawa H: **Identification of a gene expression signature associated with pediatric AML prognosis**. *Blood* 2003, **102**(5):1849-1856.

128. Ye Q-H, Qin L-X, Forgues M, He P, Kim JW, Peng AC, Simon R, Li Y, Robles AI, Chen Y *et al*: **Predicting hepatitis B virus-positive metastatic hepatocellular carcinomas using gene expression profiling and supervised machine learning**. *Nature medicine* 2003, **9**(4):416-423.

129. Beer DG, Kardia SLR, Huang C-C, Giordano TJ, Levin AM, Misek DE, Lin L, Chen G, Gharib TG, Thomas DG *et al*: **Gene-expression profiles predict survival of patients with lung adenocarcinoma**. *Nat Med* 2002, **8**(8):816-824.

130. Jones MH, Virtanen C, Honjoh D, Miyoshi T, Satoh Y, Okumura S, Nakagawa K, Nomura H, Ishikawa Y: **Two prognostically significant subtypes of high-grade lung neuroendocrine tumours independent of small-cell and large-cell neuroendocrine carcinomas identified by gene expression profiles**. *Lancet* 2004, **363**(9411):775-781.

131. Rosenwald A, Wright G, Wiestner A, Chan WC, Connors JM, Campo E, Gascoyne RD, Grogan TM, Muller-Hermelink HK, Smeland EB *et al*: **The proliferation gene expression signature is a quantitative integrator of oncogenic events that predicts survival in mantle cell lymphoma**. *Cancer cell* 2003, **3**(2):185-197.

132. Dave SS, Wright G, Tan B, Rosenwald A, Gascoyne RD, Chan WC, Fisher RI, Braziel RM, Rimsza LM, Grogan TM *et al*: **Prediction of survival in follicular lymphoma based on molecular features of tumor-infiltrating immune cells**. *N Engl J Med* 2004, **351**(21):2159-2169.

133. Rosenwald A, Wright G, Chan WC, Connors JM, Campo E, Fisher RI, Gascoyne RD, Muller-Hermelink HK, Smeland EB, Giltnane JM *et al*: **The use of molecular profiling to predict survival after chemotherapy for diffuse large-B-cell lymphoma**. *N Engl J Med* 2002, **346**(25):1937-1947.

134. Shipp MA, Ross KN, Tamayo P, Weng AP, Kutok JL, Aguiar RCT, Gaasenbeek M, Angelo M, Reich M, Pinkus GS *et al*: **Diffuse large B-cell lymphoma outcome prediction by gene-expression profiling and supervised machine learning**. *Nature medicine* 2002, **8**(1):68-74.

135. MacDonald TJ, Brown KM, LaFleur B, Peterson K, Lawlor C, Chen Y, Packer RJ, Cogen P, Stephan DA: **Expression profiling of medulloblastoma: PDGFRA and the RAS/MAPK pathway as therapeutic targets for metastatic disease**. *Nat Genet* 2001, **29**(2):143-152.

136. Talantov D, Mazumder A, Yu JX, Briggs T, Jiang Y, Backus J, Atkins D, Wang Y: **Novel genes associated with malignant melanoma but not benign melanocytic lesions**. *Clin Cancer Res* 2005, **11**(20):7234-7242.

137. Winnepenninckx V, Lazar V, Michiels S, Dessen P, Stas M, Alonso SR, Avril M-F, Ortiz Romero PL, Robert T, Balacescu O *et al*: **Gene expression profiling of primary cutaneous melanoma and clinical outcome**. *Journal of the National Cancer Institute* 2006, **98**(7):472-482.

138. Gordon GJ, Rockwell GN, Jensen RV, Rheinwald JG, Glickman JN, Aronson JP, Pottorf BJ, Nitz MD, Richards WG, Sugarbaker DJ *et al*: **Identification of novel candidate oncogenes and tumor suppressors in malignant pleural mesothelioma using large-scale transcriptional profiling**. *Am J Pathol* 2005, **166**(6):1827-1840.

139. Peters D, Freund J, Ochs RL: **Genome-wide transcriptional analysis of carboplatin response in chemosensitive and chemoresistant ovarian cancer cells**. *Mol Cancer Ther* 2005, **4**(10):1605-1616.

140. Ishikawa M, Yoshida K, Yamashita Y, Ota J, Takada S, Kisanuki H, Koinuma K, Choi YL, Kaneda R, Iwao T *et al*: **Experimental trial for diagnosis of pancreatic ductal carcinoma based on gene expression profiles of pancreatic ductal cells**. *Cancer Sci* 2005, **96**(7):387-393.

141. Dhanasekaran SM, Barrette TR, Ghosh D, Shah R, Varambally S, Kurachi K, Pienta KJ, Rubin MA, Chinnaiyan AM: **Delineation of prognostic biomarkers in prostate cancer**. *Nature* 2001, **412**(6849):822-826.

142. Lapointe J, Li C, Higgins JP, van de Rijn M, Bair E, Montgomery K, Ferrari M, Egevad L, Rayford W, Bergerheim U *et al*: **Gene expression profiling identifies clinically relevant subtypes of prostate cancer**. *Proc Natl Acad Sci U S A* 2004, **101**(3):811-816.

143. Nanni S, Priolo C, Grasselli A, D'Eletto M, Merola R, Moretti F, Gallucci M, De Carli P, Sentinelli S, Cianciulli AM *et al*: **Epithelial-restricted gene profile of primary cultures from human prostate tumors: a molecular approach to predict clinical behavior of prostate cancer**. *Mol Cancer Res* 2006, **4**(2):79-92.

144. Singh D, Febbo PG, Ross K, Jackson DG, Manola J, Ladd C, Tamayo P, Renshaw AA, D'Amico AV, Richie JP *et al*: **Gene expression correlates of clinical prostate cancer behavior**. *Cancer Cell* 2002, **1**(2):203-209.

145. Varambally S, Yu J, Laxman B, Rhodes DR, Mehra R, Tomlins SA, Shah RB, Chandran U, Monzon FA, Becich MJ *et al*: **Integrative genomic and proteomic analysis of prostate cancer reveals signatures of metastatic progression**. *Cancer cell* 2005, **8**(5):393-406.

146. Boer JM, Huber WK, Sultmann H, Wilmer F, von Heydebreck A, Haas S, Korn B, Gunawan B, Vente A, Fuzesi L *et al*: **Identification and classification of differentially expressed genes in renal cell carcinoma by expression profiling on a global human 31,500-element cDNA array**. *Genome Res* 2001, **11**(11):1861-1870.

147. Lenburg ME, Liou LS, Gerry NP, Frampton GM, Cohen HT, Christman MF: **Previously unidentified changes in renal cell carcinoma gene expression identified by parametric analysis of microarray data**. *BMC cancer* 2003, **3**:31.

148. Vasselli JR, Shih JH, Iyengar SR, Maranchie J, Riss J, Worrell R, Torres-Cabala C, Tabios R, Mariotti A, Stearman R *et al*: **Predicting survival in patients with metastatic kidney cancer by gene-expression profiling in the primary tumor**. *Proceedings of the National Academy of Sciences of the United States of America* 2003, **100**(12):6958-6963.

149. Yang XJ, Tan M-H, Kim HL, Ditlev JA, Betten MW, Png CE, Kort EJ, Futami K, Furge KA, Takahashi M *et al*: **A molecular classification of papillary renal cell carcinoma**. *Cancer Res* 2005, **65**(13):5628-5637.

150. West RB, Nuyten DSA, Subramanian S, Nielsen TO, Corless CL, Rubin BP, Montgomery K, Zhu S, Patel R, Hernandez-Boussard T *et al*: **Determination of stromal signatures in breast carcinoma**. *PLoS Biol* 2005, **3**(6):e187.

151. Detwiller KY, Fernando NT, Segal NH, Ryeom SW, D'Amore PA, Yoon SS: **Analysis of hypoxia-related gene expression in sarcomas and effect of hypoxia on RNA interference of vascular endothelial cell growth factor A**. *Cancer Res* 2005, **65**(13):5881-5889.

152. Reyes I, Iacob C, Chang Y, Suslina N, Policastro A, Moscatello A, Schantz S, Schaefer S, Tiwari R, Geliebter J: **Identification of kallikrein 7, kallikrein 10 and secreted frizzled-related protein 2 as candidate molecular markers for papillary thyroid carcinoma using microarray analysis [abstract]**. In: *the 96th Annual Meeting of the American Association for Cancer Research: 2005; Anaheim, CA*.

153. Hoffman PJ, Milliken DB, Gregg LC, Davis RR, Gregg JP: **Molecular characterization of uterine fibroids and its implication for underlying mechanisms of pathogenesis**. *Fertil Steril* 2004, **82**(3):639-649.

154. Quade BJ, Wang T-Y, Sornberger K, Dal Cin P, Mutter GL, Morton CC: **Molecular pathogenesis of uterine smooth muscle tumors from transcriptional profiling**. *Genes Chromosomes Cancer* 2004, **40**(2):97-108.
