# Supplementary material for: Computational analysis of expression of human embryonic stem cell-associated signatures in tumors
Source: BMC Res Notes. 2011 Oct 31;4:471. doi: 10.1186/1756-0500-4-471 (PMC3217937; doi:10.1186/1756-0500-4-471)
Supplement: Additional file 6 — Figure legends. The list of legends for Figure 1, 2, 3 and S1-9. [file 1756-0500-4-471-S6.DOC]

## Figure 1

Brain 3: common tumor cell vs pseudopalisading cell (Dong S et al); Brain 4: tumor vs normal (Sun L et al); Brain 5: GBM vs normal (Liang Y et al); Cervical: cancer vs normal (Wong Y.F et al); Testicular germ cell: tumor malignant vs not (Skotheim R.I et al); Esophagus 1: esophageal adenocarcinoma vs normal (Kimchi E.T et al); Esophagus 2: Barrett's esophagus vs normal (Kimchi E.T et al); Esophagus 3: esophageal epithelium tumor vs normal (Kimchi E.T et al); Gastric 1: tumor vs normal (Hippo Y et al); Gastric 2: tumor vs normal (Chen X et al); Hypopharyngeal: cancer early stage vs normal (Cromer A et al); Lung 1: adenocarcinoma vs normal (Beer D.G et al); Lung 2: adenocarcinoma vs normal (Jones M.H et al); Melanoma: malignant vs not (Talantov D et al); Pleural mesothelioma: Malignant pleural mesothelioma vs normal (Gordon G.J et al); Pancreas: Pancreatic ductal carcinoma vs normal (Ishikawa M et al); Prostate 1: cancer vs normal (Dhanasekaran S.M et al); Prostate 2: tumor vs normal (Nanni S et al); Prostate 3: tumor vs normal (Singh D et al); Prostate 4: tumor vs normal (Varambally S et al); Prostate 5: tumor vs normal (Lapointe J et al); Renal tumor 1: carcinoma vs normal (Boer J.M et al); Renal tumor 2: carcinoma vs normal (Lenburg M.E et al); Breast 1: carcinoma solitary fibrous tumor vs desmoid-type fibromatosis (West R.B et al); Sarcoma: Sarcoma vs normal (Detwiller K.Y et al); Papillary thyroid: cancer vs normal (Reyes I et al); Uterine tumor 1: Leiomyoma vs normal (Hoffman P.J et al); Uterine tumor 2: Uterine leiomyoma vs normal myometrium (Quade B.J et al); Uterine tumor 3: leiomyosarcoma vs normal myometrium (Quade B.J et al).

## Figure 2

Bladder: tumor stage T1 vs T2+ vs Ta (Dyrskjot L et al); Brain: subtype Classic vs Desmoplastic (Pomeroy S.L et al); Brain 2: *primary tumor vs recurrent tumor* (Bredel M et al 2006); Brain 3: brain tumor drug sensitive vs resistance (Bredel M et al 2006); Breast 1: tumor drug sensitive vs resistance (Chang J.C et al); Breast 2: cancer- recurred vs disease-free (Ma X.-J et al); Breast 3: tumor metastasis vs not (Sorlie T et al); Breast 4: ER alpha category IHC positive vs negative (Sotiriou C et al); Breast 5: ER category LBA positive vs negative (Sotiriou C et al); Breast 6: tumor relapse vs no (Sotiriou C et al); Breast 7: tumor metastasis vs not (van 't Veer L.J et al); Colon: tumor microsatellite instability minus vs positive (Koinuma K et al); Esophagus 4: Esophageal adenocarcinoma vs Barrett's esophagus (Kimchi E.T et al); Gastric: tumor metastasis vs no (Chen X et al); Head_Neck: head and neck squamous cell carcinomas lymph node metastasis vs no (Chung C.H et al); Hypopharyngeal: cancer metastasis vs no (Cromer A et al); Oral_Cavity: oral cavity squamous cell carcinoma metastasis vs not (O'Donnell R.K et al); CML: chronic myeloid leukemia drug responsive vs resistance (Crossman L.C et al); B-CLL: B-cell chronic lymphocytic leukemia indolent vs progressive (Falt S et al); AML: pediatric AML remission vs relapse (Yagi T et al); HCC: hepatocellular carcinomas metastasis vs no (Ye Q.-H et al); Lung 1: adenocarcinoma differentiation poor vs moderate vs well (Beer D.G et al); Lung 2: adenocarcinoma stage 1 vs stage 3 (Beer D.G et al); Lymphoma: Follicular lymphoma stage (Dave S.S et al); DLBCL 1: subgroup (Rosenwald A et al 2002); DLBCL 2: prognosis poor vs good (Shipp M.A et al); Medulloblastoma: metastatic vs not (MacDonald T.J et al); Melanoma: primary cutaneous melanoma distant metastasis vs no (Winnepenninckx V et al); Ovarian_Cancer: cancer cells chemosensitive vs chemoresistant (Peters D.J et al); Prostate 1: cancer metastasis vs no (Dhanasekaran S.M et al); Prostate 2: cancer metastasis vs no (Varambally S et al); Prostate 3: tumor metastatis vs no (Lapointe J et al); Renal 1: carcinoma metastasis vs no (Boer J.M et al); Renal 2: renal cell carcinoma histologic type 1vs type 2 (Yang X.J et al); Renal 3: renal cell carcinoma molecular type 1 vs type 2 (Yang X.J et al); Uterine: leiomyoma vs uterine leiomyosarcoma (Quade B.J et al).

## Figure 3

Brain: Brain tumor (Pomeroy S.L et al); Gastric: Gastric tumor (Chen X et al); lymphoma 1: Mantle cell lymphoma (Rosenwald A et al 2003); lymphoma 2: Follicular lymphoma (Dave S.S et al); DLBCL: diffuse large-B-cell lymphoma (Rosenwald A et al 2002).

**Figure S1**

Brain 3: common tumor cell vs pseudopalisading cell (Dong S et al); Brain 4: tumor vs normal (Sun L et al); Brain 5: GBM vs normal (Liang Y et al); Cervical: cancer vs normal (Wong Y.F et al); Testicular germ cell: tumor malignant vs not (Skotheim R.I et al); Esophagus 1: esophageal adenocarcinoma vs normal (Kimchi E.T et al); Esophagus 2: Barrett's esophagus vs normal (Kimchi E.T et al); Esophagus 3: esophageal epithelium tumor vs normal (Kimchi E.T et al); Gastric 1: tumor vs normal (Hippo Y et al); Gastric 2: tumor vs normal (Chen X et al); Hypopharyngeal: cancer early stage vs normal (Cromer A et al); Lung 1: adenocarcinoma vs normal (Beer D.G et al); Melanoma: malignant vs not (Talantov D et al); Pleural mesothelioma: Malignant pleural mesothelioma vs normal (Gordon G.J et al); Pancreas: Pancreatic ductal carcinoma vs normal (Ishikawa M et al); Prostate 1: cancer vs normal (Dhanasekaran S.M et al); Prostate 2: tumor vs normal (Nanni S et al); Prostate 3: tumor vs normal (Singh D et al); Prostate 4: tumor vs normal (Varambally S et al); Prostate 5: tumor vs normal (Lapointe J et al); Renal tumor 1: carcinoma vs normal (Boer J.M et al); Renal tumor 2: carcinoma vs normal (Lenburg M.E et al); Breast 1: carcinoma solitary fibrous tumor vs desmoid-type fibromatosis (West R.B et al); Sarcoma: Sarcoma vs normal (Detwiller K.Y et al); Papillary thyroid: cancer vs normal (Reyes I et al); Uterine tumor 1: Leiomyoma vs normal (Hoffman P.J et al); Uterine tumor 2: Uterine leiomyoma vs normal myometrium (Quade B.J et al); Uterine tumor 3: leiomyosarcoma vs normal myometrium (Quade B.J et al).

**Figure S2**

Bladder: tumor stage T1 vs T2+ vs Ta (Dyrskjot L et al); Brain: subtype Classic vs Desmoplastic (Pomeroy S.L et al); Breast 1: tumor drug sensitive vs resistance (Chang J.C et al); Breast 2: cancer- recurred vs disease-free (Ma X.-J et al); Breast 3: tumor metastasis vs not (Sorlie T et al); Breast 4: ER alpha category IHC positive vs negative (Sotiriou C et al); Breast 5: ER category LBA positive vs negative (Sotiriou C et al); Breast 6: tumor relapse vs no (Sotiriou C et al); Breast 7: tumor metastasis vs not (van 't Veer L.J et al); Colon: tumor microsatellite instability minus vs positive (Koinuma K et al); Esophagus 4: Esophageal adenocarcinoma vs Barrett's esophagus (Kimchi E.T et al); Gastric: tumor metastasis vs no (Chen X et al); Head_Neck: head and neck squamous cell carcinomas lymph node metastasis vs no (Chung C.H et al); Hypopharyngeal: cancer metastasis vs no (Cromer A et al); Oral_Cavity: oral cavity squamous cell carcinoma metastasis vs not (O'Donnell R.K et al); CML: chronic myeloid leukemia drug responsive vs resistance (Crossman L.C et al); B-CLL: B-cell chronic lymphocytic leukemia indolent vs progressive (Falt S et al); AML: pediatric AML remission vs relapse (Yagi T et al); HCC: hepatocellular carcinomas metastasis vs no (Ye Q.-H et al); Lung 1: adenocarcinoma differentiation poor vs moderate vs well (Beer D.G et al); Lung 2: adenocarcinoma stage 1 vs stage 3 (Beer D.G et al); DLBCL 1: subgroup (Rosenwald A et al 2002); DLBCL 2: prognosis poor vs good (Shipp M.A et al); Medulloblastoma: metastatic vs not (MacDonald T.J et al); Melanoma: primary cutaneous melanoma distant metastasis vs no (Winnepenninckx V et al); Ovarian_Cancer: cancer cells chemosensitive vs chemoresistant (Peters D.J et al); Prostate 1: cancer metastasis vs no (Dhanasekaran S.M et al); Prostate 2: cancer metastasis vs no (Varambally S et al); Prostate 3: tumor metastatis vs no (Lapointe J et al); Renal 1: carcinoma metastasis vs no (Boer J.M et al); Renal 2: renal cell carcinoma histologic type 1 vs type 2 (Yang X.J et al); Renal 3: renal cell carcinoma molecular type 1 vs type 2 (Yang X.J et al); Uterine: leiomyoma vs uterine leiomyosarcoma (Quade B.J et al).

**Figure S3**

Brain: Brain tumor (Pomeroy S.L et al); Gastric: Gastric tumor (Chen X et al); lymphoma 1: Mantle cell lymphoma (Rosenwald A et al 2003); lymphoma 2: Follicular lymphoma (Dave S.S et al); DLBCL: diffuse large-B-cell lymphoma (Rosenwald A et al 2002); Kidney: Metastatic kidney cancer (Vasselli J.R et al).

**Figure S4**

Brain 1: tumor vs normal (Bredel M et al 2005); Brain 2: normal vs oligodendroglioma (Bredel M et al 2005); Brain 3: common tumor cell vs pseudopalisading cell (Dong S et al); Brain 4: tumor vs normal (Sun L et al); Brain 5: GBM vs normal (Liang Y et al); Brain 6: normal vs glioblastoma (Bredel M et al 2005); Cervical: cancer vs normal (Wong Y.F et al); Testicular germ cell: tumor malignant vs not (Skotheim R.I et al); Esophagus 1: esophageal adenocarcinoma vs normal (Kimchi E.T et al); Esophagus 2: Barrett's esophagus vs normal (Kimchi E.T et al); Esophagus 3: esophageal epithelium tumor vs normal (Kimchi E.T et al); Gastric 1: tumor vs normal (Hippo Y et al); Gastric 2: tumor vs normal (Chen X et al); Hypopharyngeal: cancer early stage vs normal (Cromer A et al); Lung 1: adenocarcinoma vs normal (Beer D.G et al); Lung 2: adenocarcinoma vs normal (Jones M.H et al); Melanoma: malignant vs not (Talantov D et al); Pleural mesothelioma: Malignant pleural mesothelioma vs normal (Gordon G.J et al); Pancreas: Pancreatic ductal carcinoma vs normal (Ishikawa M et al); Prostate 1: cancer vs normal (Dhanasekaran S.M et al); Prostate 2: tumor vs normal (Nanni S et al); Prostate 3: tumor vs normal (Singh D et al); Prostate 4: tumor vs normal (Varambally S et al); Prostate 5: tumor vs normal (Lapointe J et al); Renal tumor 1: carcinoma vs normal (Boer J.M et al); Renal tumor 2: carcinoma vs normal (Lenburg M.E et al); Breast 1: carcinoma solitary fibrous tumor vs desmoid-type fibromatosis (West R.B et al); Sarcoma: Sarcoma vs normal (Detwiller K.Y et al); Papillary thyroid: cancer vs normal (Reyes I et al); Uterine tumor 1: Leiomyoma vs normal (Hoffman P.J et al); Uterine tumor 2: Uterine leiomyoma vs normal myometrium (Quade B.J et al); Uterine tumor 3: leiomyosarcoma vs normal myometrium (Quade B.J et al).

**Figure S5**

Bladder: tumor stage T1 vs T2+ vs Ta (Dyrskjot L et al); Brain: subtype Classic vs Desmoplastic (Pomeroy S.L et al); Breast 1: tumor drug sensitive vs resistance (Chang J.C et al); Breast 2: cancer- recurred vs disease-free (Ma X.-J et al); Breast 3: tumor metastasis vs not (Sorlie T et al); Breast 4: ER alpha category IHC positive vs negative (Sotiriou C et al); Breast 5: ER category LBA positive vs negative (Sotiriou C et al); Breast 6: tumor relapse vs no (Sotiriou C et al); Breast 7: tumor metastasis vs not (van 't Veer L.J et al); Colon: tumor microsatellite instability minus vs positive (Koinuma K et al); Esophagus 4: Esophageal adenocarcinoma vs Barrett's esophagus (Kimchi E.T et al); Gastric: tumor metastasis vs no (Chen X et al); Head_Neck: head and neck squamous cell carcinomas lymph node metastasis vs no (Chung C.H et al); Hypopharyngeal: cancer metastasis vs no (Cromer A et al); Oral_Cavity: oral cavity squamous cell carcinoma metastasis vs not (O'Donnell R.K et al); CML: chronic myeloid leukemia drug responsive vs resistance (Crossman L.C et al); B-CLL: B-cell chronic lymphocytic leukemia indolent vs progressive (Falt S et al); AML: pediatric AML remission vs relapse (Yagi T et al); HCC: hepatocellular carcinomas metastasis vs no (Ye Q.-H et al); Lung 1: adenocarcinoma differentiation poor vs moderate vs well (Beer D.G et al); Lung 2: adenocarcinoma stage 1 vs stage 3 (Beer D.G et al); Lymphoma: Follicular lymphoma stage (Dave S.S et al); DLBCL 1: subgroup (Rosenwald A et al 2002); DLBCL 2: prognosis poor vs good (Shipp M.A et al); Medulloblastoma: metastatic vs not (MacDonald T.J et al); Melanoma: primary cutaneous melanoma distant metastasis vs no (Winnepenninckx V et al); Ovarian_Cancer: cancer cells chemosensitive vs chemoresistant (Peters D.J et al); Prostate 1: cancer metastasis vs no (Dhanasekaran S.M et al); Prostate 2: cancer metastasis vs no (Varambally S et al); Prostate 5: tumor vs normal (Lapointe J et al); Renal 1: carcinoma metastasis vs no (Boer J.M et al); Renal 2: renal cell carcinoma histologic type 1 vs type 2 (Yang X.J et al); Renal 3: renal cell carcinoma molecular type 1vs type 2 (Yang X.J et al); Uterine: leiomyoma vs uterine leiomyosarcoma (Quade B.J et al).

**Figure S6**

Brain: Brain tumor (Pomeroy S.L et al); Gastric: Gastric tumor (Chen X et al); lymphoma 1: Mantle cell lymphoma (Rosenwald A et al 2003); lymphoma 2: Follicular lymphoma (Dave S.S et al); DLBCL: diffuse large-B-cell lymphoma (Rosenwald A et al 2002); Kidney: Metastatic kidney cancer (Vasselli J.R et al).

**Figure S7**

Brain 1: tumor vs normal (Bredel M et al 2005); Brain 2: normal vs oligodendroglioma (Bredel M et al 2005); Brain 3: common tumor cell vs pseudopalisading cell (Dong S et al); Brain 4: tumor vs normal (Sun L et al); Brain 5: GBM vs normal (Liang Y et al); Cervical: cancer vs normal (Wong Y.F et al); Testicular germ cell: tumor malignant vs not (Skotheim R.I et al); Gastric 1: tumor vs normal (Hippo Y et al); Gastric 2: tumor vs normal (Chen X et al); Hypopharyngeal: cancer early stage vs normal (Cromer A et al); Lung 1: adenocarcinoma vs normal (Beer D.G et al); Lung 2: adenocarcinoma vs normal (Jones M.H et al); Melanoma: malignant vs not (Talantov D et al); Pleural mesothelioma: Malignant pleural mesothelioma vs normal (Gordon G.J et al); Pancreas: Pancreatic ductal carcinoma vs normal (Ishikawa M et al); Prostate 1: cancer vs normal (Dhanasekaran S.M et al); Prostate 2: tumor vs normal (Nanni S et al); Prostate 3: tumor vs normal (Singh D et al); Prostate 4: tumor vs normal (Varambally S et al); Renal tumor 1: carcinoma vs normal (Boer J.M et al); Renal tumor 2: carcinoma vs normal (Lenburg M.E et al); Breast 1: carcinoma solitary fibrous tumor vs desmoid-type fibromatosis (West R.B et al); Sarcoma: Sarcoma vs normal (Detwiller K.Y et al); Papillary thyroid: cancer vs normal (Reyes I et al); Uterine tumor 1: Leiomyoma vs normal (Hoffman P.J et al); Uterine tumor 2: Uterine leiomyoma vs normal myometrium (Quade B.J et al); Uterine tumor 3: leiomyosarcoma vs normal myometrium (Quade B.J et al).

**Figure S8.**

Bladder: tumor stage T1 vs T2+ vs Ta (Dyrskjot L et al); Brain: subtype Classic vs Desmoplastic (Pomeroy S.L et al); Breast 1: tumor drug sensitive vs resistance (Chang J.C et al); Breast 2: cancer- recurred vs disease-free (Ma X.-J et al); Breast 3: tumor metastasis vs not (Sorlie T et al); Breast 4: ER alpha category IHC positive vs negative (Sotiriou C et al); Breast 5: ER category LBA positive vs negative (Sotiriou C et al); Breast 6: tumor relapse vs no (Sotiriou C et al); Breast 7: tumor metastasis vs not (van 't Veer L.J et al); Colon: tumor microsatellite instability minus vs positive (Koinuma K et al); Gastric: tumor metastasis vs no (Chen X et al); Head_Neck: head and neck squamous cell carcinomas lymph node metastasis vs no (Chung C.H et al); Hypopharyngeal: cancer metastasis vs no (Cromer A et al); Oral_Cavity: oral cavity squamous cell carcinoma metastasis vs not (O'Donnell R.K et al); CML: chronic myeloid leukemia drug responsive vs resistance (Crossman L.C et al); B-CLL: B-cell chronic lymphocytic leukemia indolent vs progressive (Falt S et al); AML: pediatric AML remission vs relapse (Yagi T et al); HCC: hepatocellular carcinomas metastasis vs no (Ye Q.-H et al); Lung 1: adenocarcinoma differentiation poor vs moderate vs well (Beer D.G et al); Lung 2: adenocarcinoma stage 1 vs stage 3 (Beer D.G et al); DLBCL 1: subgroup (Rosenwald A et al 2002); DLBCL 2: prognosis poor vs good (Shipp M.A et al); Medulloblastoma: metastatic vs not (MacDonald T.J et al); Melanoma: primary cutaneous melanoma distant metastasis vs no (Winnepenninckx V et al); Ovarian_Cancer: cancer cells chemosensitive vs chemoresistant (Peters D.J et al); Prostate 1: cancer metastasis vs no (Dhanasekaran S.M et al); Prostate 2: cancer metastasis vs no (Varambally S et al); Renal 1: carcinoma metastasis vs no (Boer J.M et al); Renal 2: renal cell carcinoma histologic type 1vs type 2 (Yang X.J et al); Renal 3: renal cell carcinoma molecular type 1vs type 2 (Yang X.J et al); Uterine: leiomyoma vs uterine leiomyosarcoma (Quade B.J et al).

**Figure S9**

Brain: Brain tumor (Pomeroy S.L et al); Gastric: Gastric tumor (Chen X et al); lymphoma 1: Mantle cell lymphoma (Rosenwald A et al 2003); lymphoma 2: Follicular lymphoma (Dave S.S et al); DLBCL: diffuse large-B-cell lymphoma (Rosenwald A et al 2002); Kidney: Metastatic kidney cancer (Vasselli J.R et al).
