# Supplementary figures and images for: Computational analysis of expression of human embryonic stem cell-associated signatures in tumors
Source: BMC Res Notes. 2011 Oct 31;4:471. doi: 10.1186/1756-0500-4-471 (PMC3217937; doi:10.1186/1756-0500-4-471)

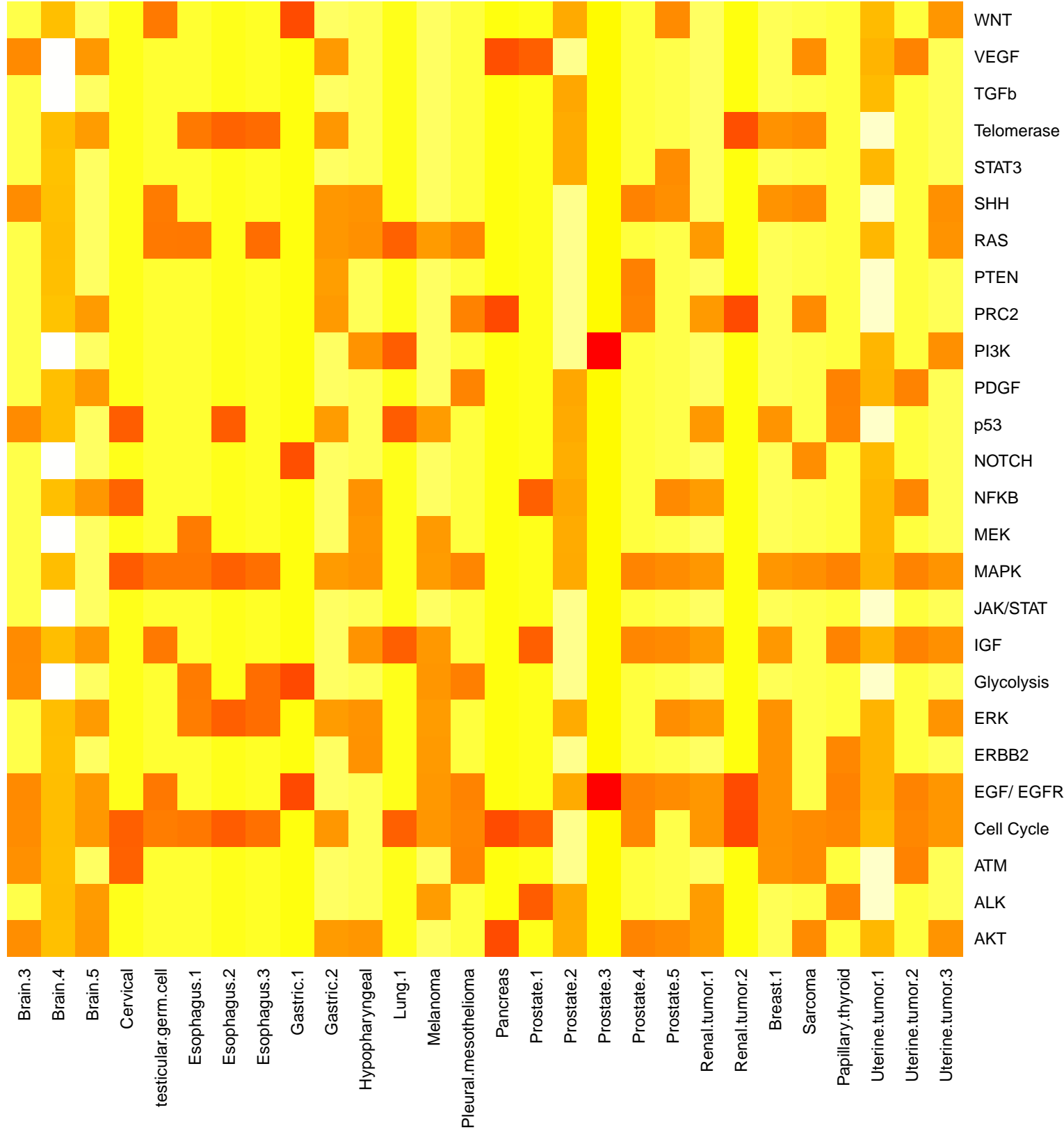

Supplement: Additional file 7 — Figure S1. Significance of overlap between hESC and tumor pathways by normal vs. tumor class comparison. The detailed description of all the datasets is provided in Additional file 6. [file 1756-0500-4-471-S7.PDF]

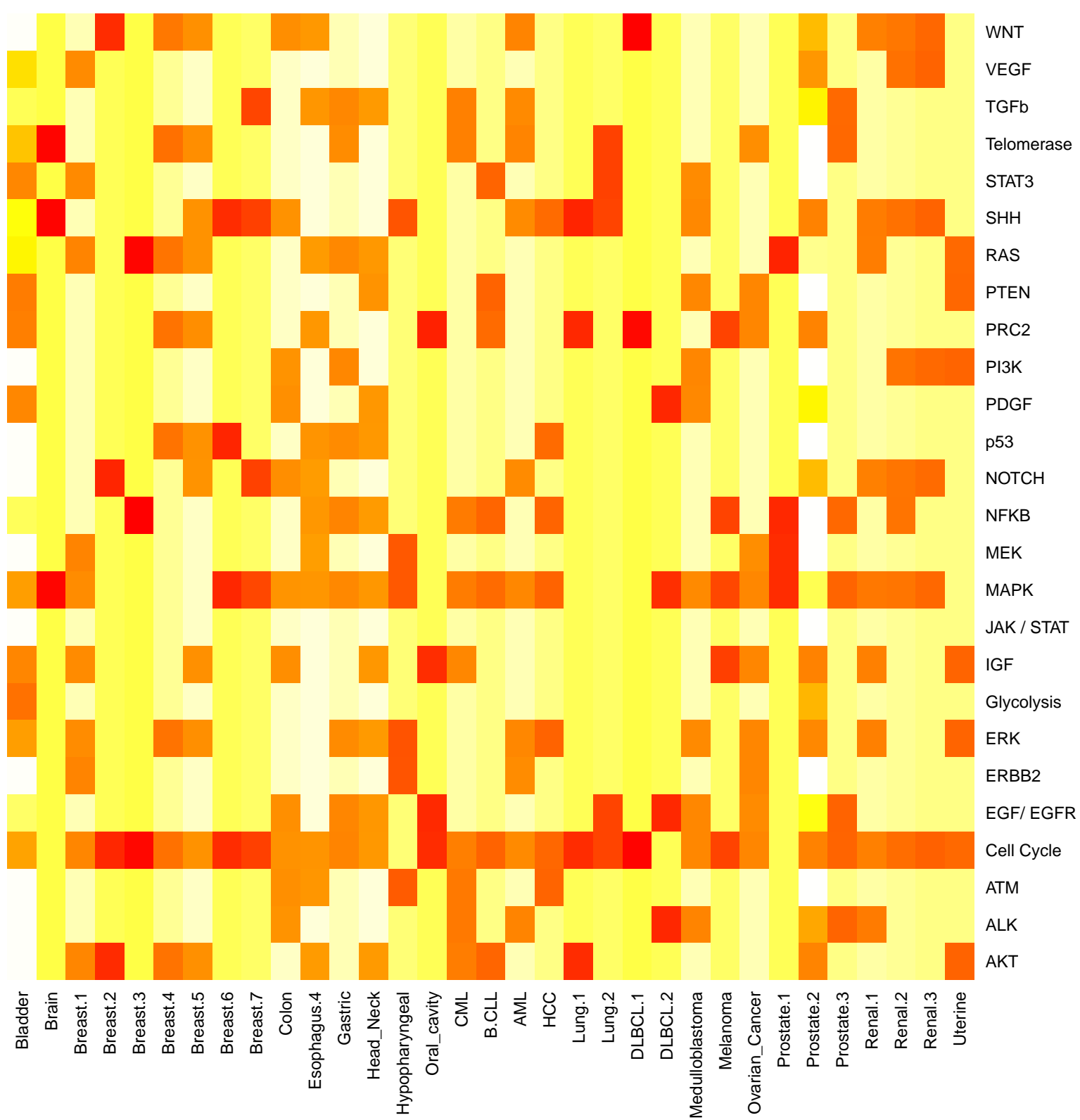

Supplement: Additional file 8 — Figure S2. Significance of overlap between hESC and tumor pathways by good vs. poor prognosis class comparison. The detailed description of all the datasets is provided in Additional file 6. [file 1756-0500-4-471-S8.PDF]

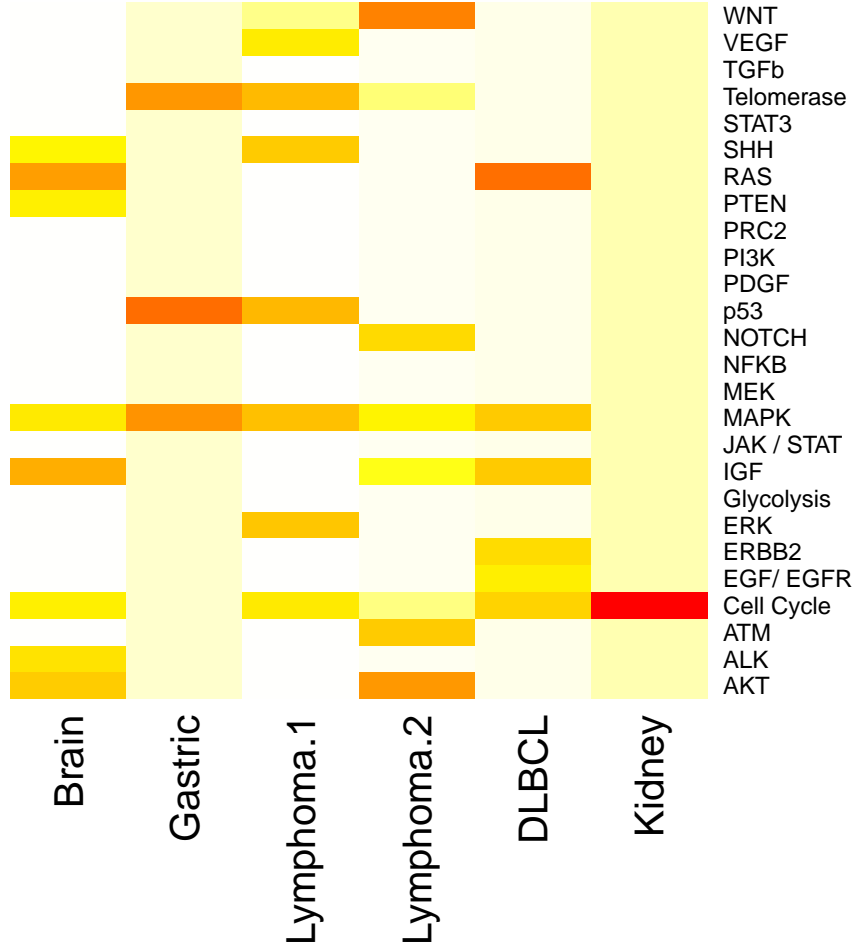

Supplement: Additional file 9 — Figure S3. Significance of overlap between hESC and tumor pathways by survival analysis. The detailed description of all the datasets is provided in Additional file 6. [file 1756-0500-4-471-S9.PDF]

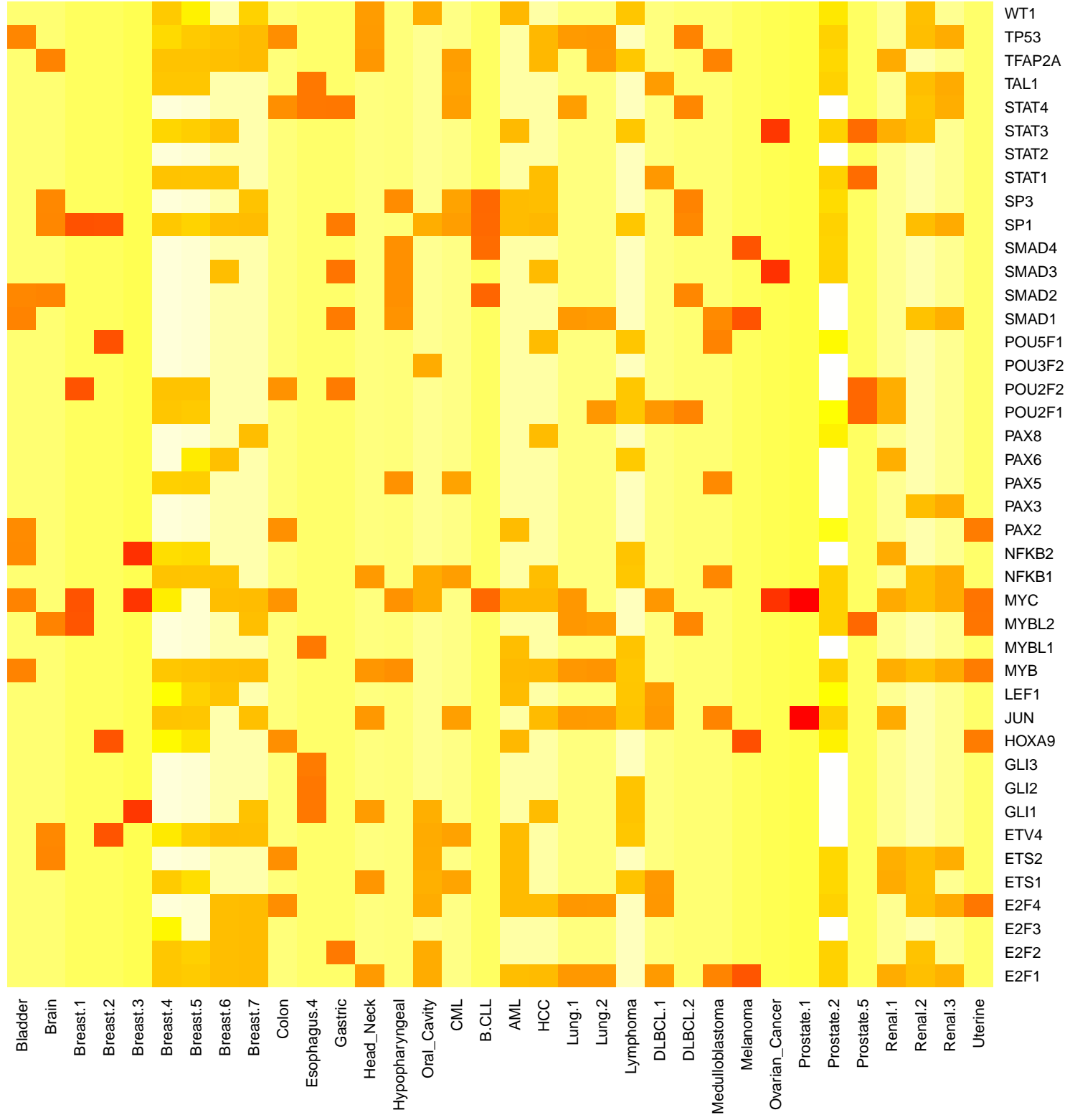

Supplement: Additional file 11 — Figure S5. Significance of overlap between hESC and tumor TFs by good vs. poor prognosis class comparison. The detailed description of all the datasets is provided in Additional file 6. [file 1756-0500-4-471-S11.PDF]

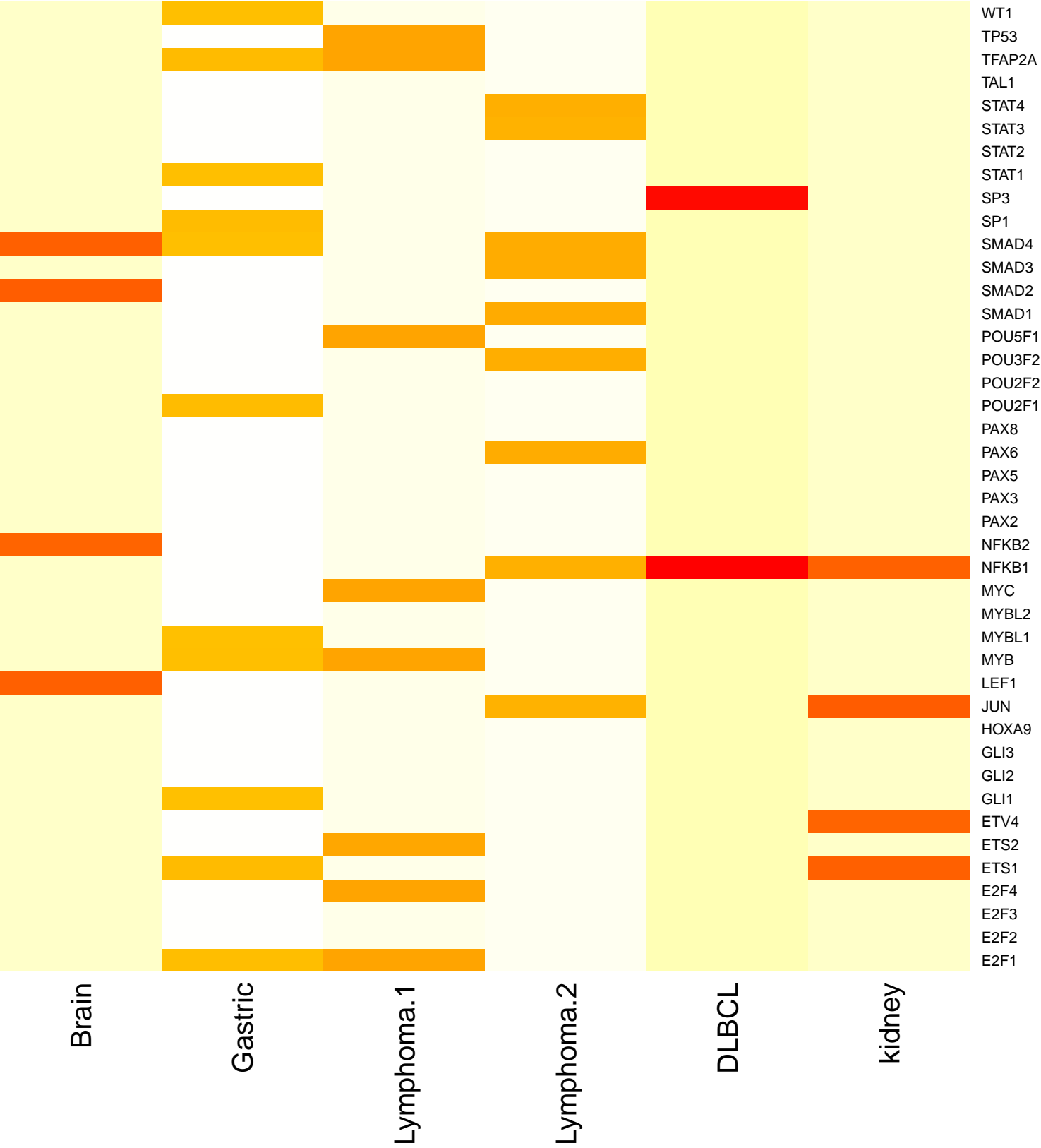

Supplement: Additional file 12 — Figure S6. Significance of overlap between hESC and tumor TFs by survival analysis. The detailed description of all the datasets is provided in Additional file 6. [file 1756-0500-4-471-S12.PDF]

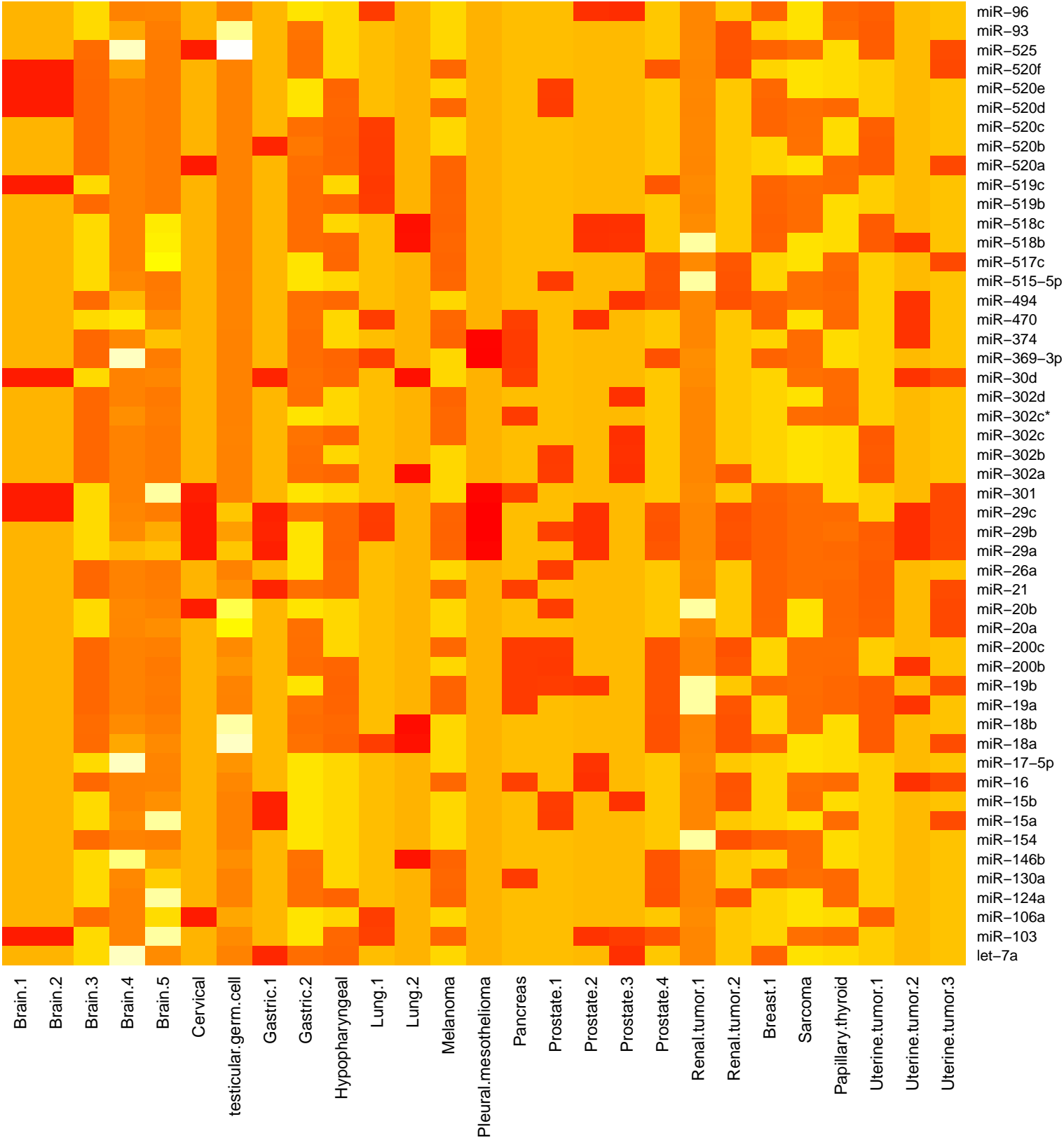

Supplement: Additional file 14 — Figure S7. Significance of overlap between hESC and tumor miRNAs by normal vs. tumor class comparison. The detailed description of all the datasets is provided in Additional file 6. [file 1756-0500-4-471-S14.PDF]

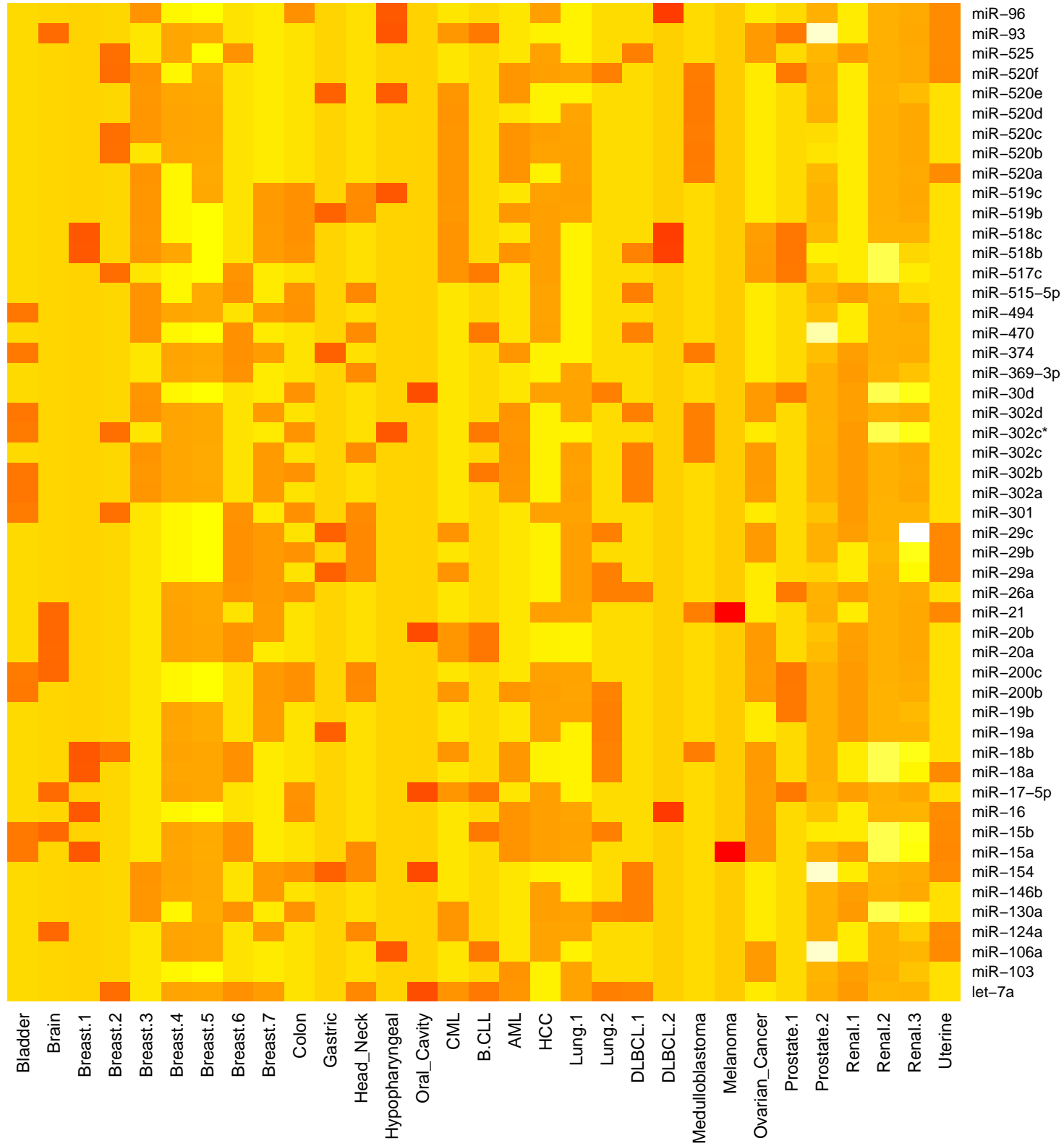

Supplement: Additional file 15 — Figure S8. Significance of overlap between hESC and tumor miRNAs by good vs. poor prognosis class comparison. The detailed description of all the datasets is provided in Additional file 6. [file 1756-0500-4-471-S15.PDF]

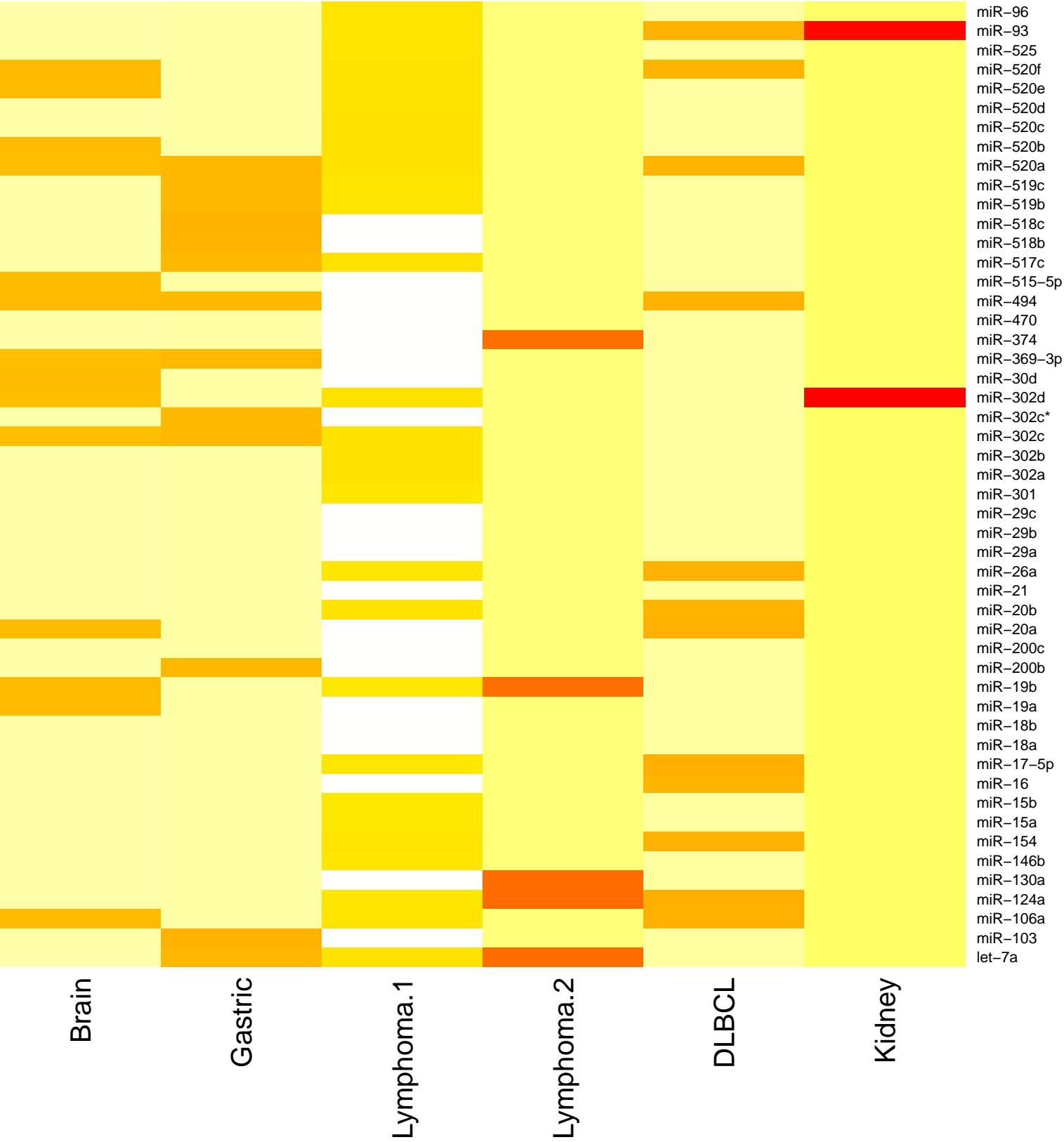

Supplement: Additional file 16 — Figure S9. Significance of overlap between hESC and tumor miRNAs by survival analysis. The detailed description of all the datasets is provided in Additional file 6. [file 1756-0500-4-471-S16.PDF]
