# Supplementary material for: Computational analysis of expression of human embryonic stem cell-associated signatures in tumors
Source: BMC Res Notes. 2011 Oct 31;4:471. doi: 10.1186/1756-0500-4-471 (PMC3217937; doi:10.1186/1756-0500-4-471)
Supplement: Additional file 10 — Figure S4. Significance of overlap between hESC and tumor TFs by normal vs. tumor class comparison. The detailed description of all the datasets is provided in Additional file 6. [file 1756-0500-4-471-S10.PDF]

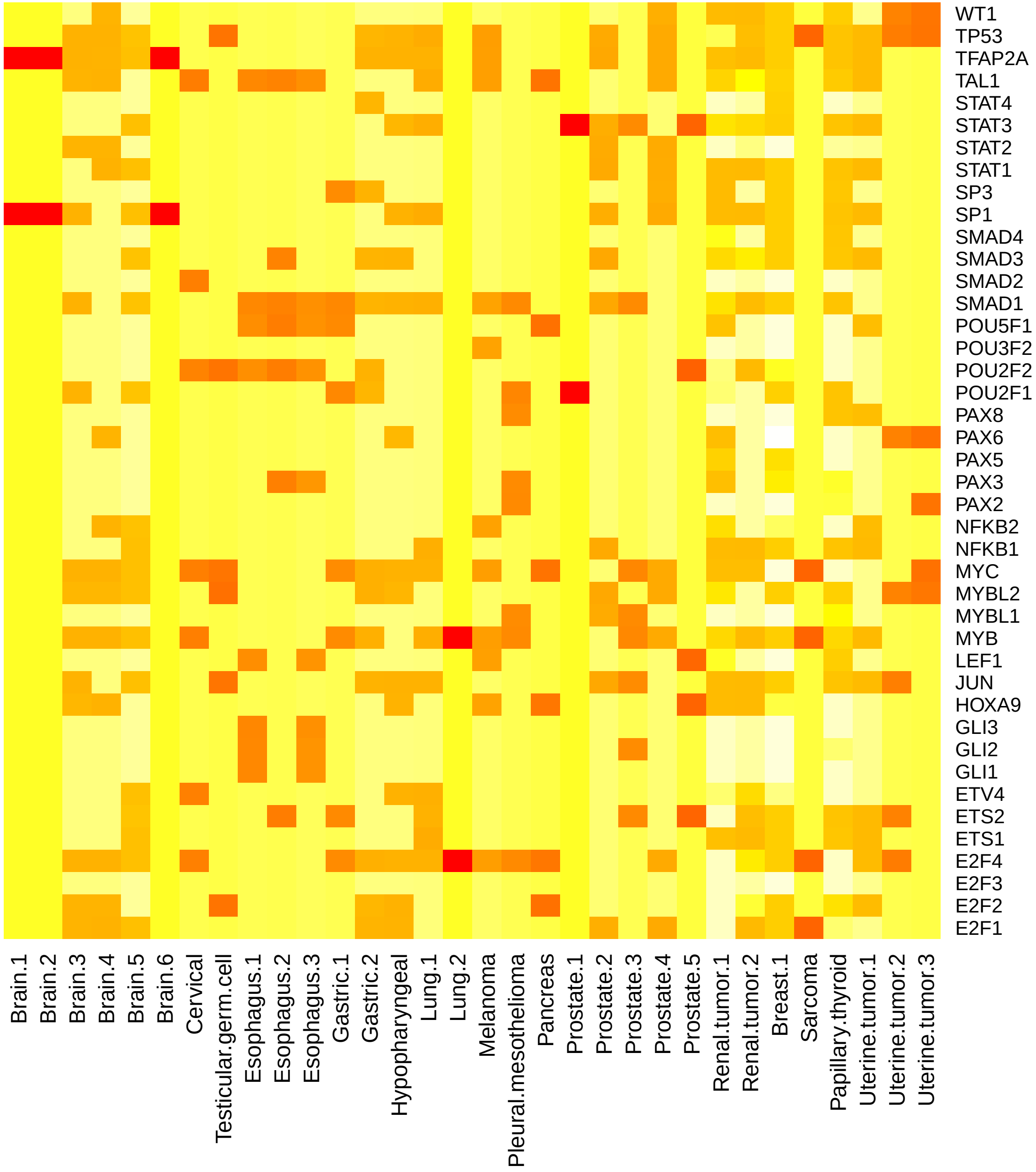

WT1
TP53
TFAP2A
TAL1
STAT4
STAT3
STAT2
STAT1
SP3
SP1
SMAD4
SMAD3
SMAD2
SMAD1
POU5F1
POU3F2
POU2F2
POU2F1
PAX8
PAX6
PAX5
PAX3
PAX2
NFKB2
NFKB1
MYC
MYBL2
MYBL1
MYB
LEF1
JUN
HOXA9
GLI3
GLI2
GLI1
ETV4
ETS2
ETS1
E2F4
E2F3
E2F2
E2F1
Brain.1
Brain.2
Brain.3
Brain.4
Brain.5
Brain.6
Cervical
Testicular.germ.cell
Esophagus.1
Esophagus.2
Esophagus.3
Gastric.1
Gastric.2
Hypopharyngeal
Lung.1
Lung.2
Melanoma
Pleural.mesothelioma
Pancreas
Prostate.1
Prostate.2
Prostate.3
Prostate.4
Prostate.5
Renal.tumor.1
Renal.tumor.2
Breast.1
Sarcoma
Papillary.thyroid
Uterine.tumor.1
Uterine.tumor.2
Uterine.tumor.3
